# Interplay of Electronic and Steric Effects to Yield Low‐Temperature CO Oxidation at Metal Single Sites in Defect‐Engineered HKUST‐1

**DOI:** 10.1002/anie.202000385

**Published:** 2020-04-17

**Authors:** Weijia Wang, Dmitry I. Sharapa, Abhinav Chandresh, Alexei Nefedov, Stefan Heißler, Lars Heinke, Felix Studt, Yuemin Wang, Christof Wöll

**Affiliations:** ^1^ Institute of Functional Interfaces (IFG) Karlsruhe Institute of Technology (KIT) 76344 Eggenstein-Leopoldshafen Germany; ^2^ Institute of Catalysis Research and Technology (IKFT) Karlsruhe Institute of Technology (KIT) 76344 Eggenstein-Leopoldshafen Germany

**Keywords:** density functional theory, dioxygen activation, MOFs, reaction mechanism, single-atom sites

## Abstract

In contrast to catalytically active metal single atoms deposited on oxide nanoparticles, the crystalline nature of metal‐organic frameworks (MOFs) allows for a thorough characterization of reaction mechanisms. Using defect‐free HKUST‐1 MOF thin films, we demonstrate that Cu^+^/Cu^2+^ dimer defects, created in a controlled fashion by reducing the pristine Cu^2+^/Cu^2+^ pairs of the intact framework, account for the high catalytic activity in low‐temperature CO oxidation. Combining advanced IR spectroscopy and density functional theory we propose a new reaction mechanism where the key intermediate is an uncharged O_2_ species, weakly bound to Cu^+^/Cu^2+^. Our results reveal a complex interplay between electronic and steric effects at defect sites in MOFs and provide important guidelines for tailoring and exploiting the catalytic activity of single metal atom sites.

## Graphical abstract

**A complex interplay**: A thorough study combining advanced spectroscopy and theory reveals that electronic and steric effects occur at reduced Cu^+^/Cu^2+^ single dimers in defect‐engineered HKUST‐1 SURMOF to yield high catalytic activity for low‐temperature CO oxidation. A new reaction mechanism is presented involving an uncharged O_2_ species as the key intermediate.

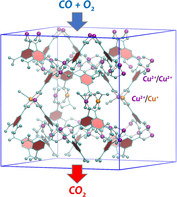

Atomically dispersed precious metals are presently receiving considerable attention in catalysis due to their unique chemical activities and large per‐atom conversion yields. Conventional systems, that is, metal particles deposited on oxides by impregnation methods, typically yield broad size distributions, thus reducing the per‐atom yield. Although the presence of single atom active sites has been demonstrated in a number of cases,^1^ the typically rather amorphous nature of the metal/oxide interface with a high heterogeneity of metal clusters and particles have made the unambiguous identification of reaction mechanisms and the accurate validation of theoretical results very difficult. In this context, metal‐organic frameworks (MOFs) offer a number of interesting features. First, many of these crystalline coordination polymers obtained by connecting metal or metal/oxo clusters via organic linkers feature coordinately unsaturated metal sites (CUS). In the MOF case, the metal atoms are prevented from sintering by strong ionic bonds to the molecular struts forming the framework. Furthermore, these metal single atom sites are located at well‐defined positions of a crystalline, porous lattice and are thus homogeneously dispersed within the MOF. Consequently, MOF‐based materials hold great promise as single‐site catalysts.^2^ However, in many cases it is unclear whether the observed catalytic activity is related to metal ions of the perfect framework, or whether the active sites are related to structural defects within the MOF. In fact, defect engineering of MOFs has been used in numerous cases to tune the structural, electronic and chemical properties of MOFs in a controlled fashion.^3^

MOF powders, the most common form of MOF materials, often exhibit large defect densities even before introducing them intentionally, and there is no case known where an unambiguous identification of active sites and the reaction mechanisms together with a fully satisfying theoretical analysis has been reported.

In order to achieve an atomic‐level understanding of single active sites and the mechanism governing a prototype reaction, low‐temperature CO oxidation, we have carried out an extensive experimental and theoretical study based on defect‐free HKUST‐1 thin films (surface‐mounted MOFs, SURMOFs) fabricated using liquid‐phase quasi‐epitaxy.^4^ The oxidation of carbon monoxide was then investigated by combining infrared reflection absorption spectroscopy (IRRAS) and quantum chemical calculations based on density functional theory (DFT) as well as complete active space (CAS) calculations to treat transition states with multi‐reference character.^5^

After synthesis of the HKUST‐1 SURMOF, the structure of the porous framework was characterized using X‐ray diffraction (XRD, Supporting Information, Figure S1) and the defect density (i.e., the concentration of Cu^+^ species) was determined by IRRAS using CO as a probe molecule. As shown in Figure 1 a, there is only one IR band at 2174 cm^−1^ characteristic for CO bound to the axial positions of pristine Cu^2+^/Cu^2+^ paddle‐wheel units (Figure 1 b).3c, 6 This data as well as the deconvoluted Cu 2p XPS results (Supporting Information, Figure S2a) allow setting an upper limit of 2 % to the concentration of the Cu^+^ species.

**Figure 1:**
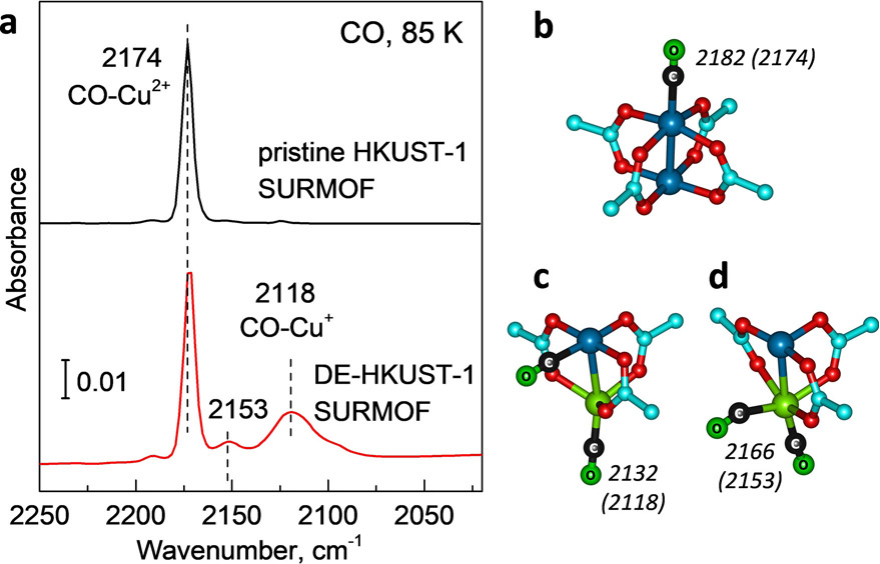
Pristine and defect‐engineered (DE)‐HKUST‐1 SURMOFs characterized by IRRAS and DFT calculations. a) IRRAS data of CO adsorption on the pristine and DE‐HKUST‐1 SURMOF at 85 K. DFT‐optimized atomic structures of CO adsorbed to b) pristine HKUST‐1 and c,d) DE‐HKUST‐1: c) CO‐Cu^+^, d) (CO)_2_‐Cu^+^, together with the computed CO stretch frequencies. For comparison, the corresponding experimental values are given in the parenthesis. C of MOF‐cyan, O of MOF‐red, Cu^2+^‐blue, Cu^+^‐green, C of CO‐black, O of CO‐lime.

For the pristine HKUST‐1 SURMOF, the activity for CO oxidation was found to be very low. In Figure S3 we show the in situ IRRAS data recorded during exposing the CO‐saturated SURMOF to O_2_ at 105 K. The intensity of the CO‐Cu^2+^ band remained nearly unchanged in oxygen atmosphere. This result is not consistent with conclusions reported in previous work,^7^ the reasons will be discussed below.

In order to unravel the role of defects in this system, we introduced Cu^+^‐containing defects in a controlled fashion by using a thermal defect‐engineering (DE) strategy.^8^ Briefly, subjecting the pristine, virtually perfect HKUST‐1 SURMOF to annealing in UHV at elevated temperatures leads to an oxidative decarboxylation, causing a reduction of the Cu^2+^/Cu^2+^ paddle‐wheel units, thus yielding Cu^+^/Cu^2+^ defect pairs. A thorough XPS analysis yields a Cu^+^ concentration of 30 % created by heating the pristine MOFs at 430 K for 30 min (Supporting Information, Figure S2 b). The IRRAS data, obtained after exposure of these DE‐HKUST‐1 SURMOFs to CO, clearly reveal a vibrational band at 2118 cm^−1^ (Figure 1 a). This band, red‐shifted by 56 cm^−1^ relative to CO bound to Cu^2+^ species within a perfect paddle wheel, is characteristic for CO ligated to Cu^+^ species.3c, 6 Figure 1 c shows the DFT‐optimized atomic structure of CO species bound to Cu^+^/Cu^2+^ dimer defects. The computed red shift of CO‐Cu^+^ vs. CO‐Cu^2+^ (50 cm^−1^) is in excellent agreement with the experimental results.

Importantly, the loss of the carboxylate group reducing one of the Cu ions to yield Cu^+^/Cu^2+^ creates additional open space. Based on the DFT calculations, a second CO can bind to the Cu^+^ ion, see Figure 1 d. Accordingly, the band at 2153 cm^−1^ (Figure 1 a) is assigned to the asymmetric stretching mode of geminal (CO)_2_ dimers at the Cu^+^ CUS sites. As shown in Figure 2 a and 2 b, the DE‐HKUST‐1 SURMOF exhibits pronounced catalytic activity for the CO oxidation reaction. Even at low temperatures of 105 K, exposure to O_2_ leads to a substantial reduction of the CO‐Cu^2+^ vibrational band at 2180 cm^−1^. While the CO_2_ product was observed inside HKUST‐1 powders,^7^ for the very thin (100 nm) SURMOFs the amount was too small to be detected.

**Figure 2:**
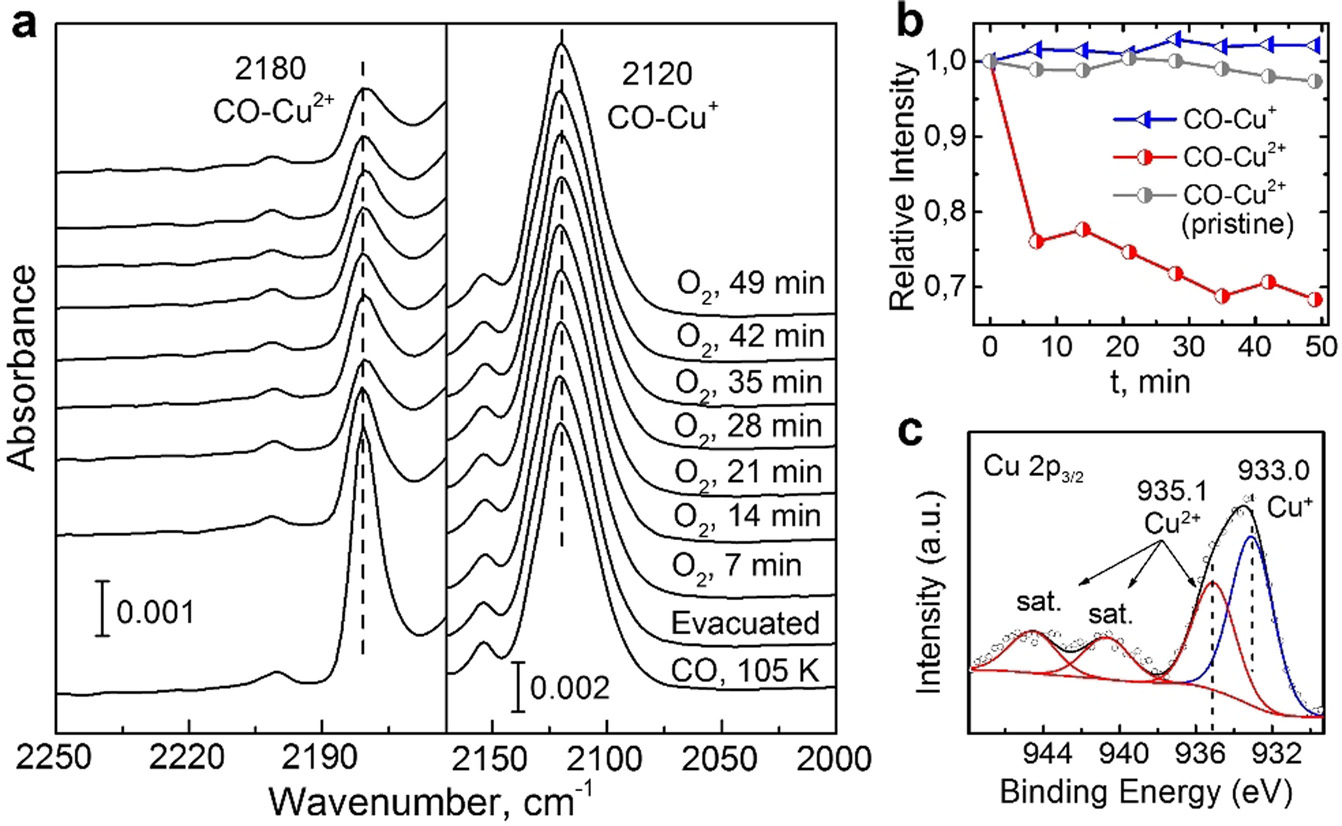
Low‐temperature CO oxidation on DE‐HKUST‐1 SURMOF. a) IRRAS data recorded during exposing the DE‐HKUST‐1 SURMOF first to CO and then to O_2_ (10^−5^ mbar) for different times at 105 K. b) Integrated intensity evolution of the two spectral components: the CO‐Cu^+^ band at 2120 cm^−1^ (blue) and the CO‐Cu^2+^ band at 2180 cm^−1^ (red). For comparison, the results for the pristine HKUST‐1 are also shown (gray). c) Cu 2p_3/2_ XPS data of DE‐HKUST‐1 SURMOF.

On the basis of these observations, we propose that the catalytic activity of HKUST‐1 for CO oxidation results from the presence of reduced Cu^+^/Cu^2+^ dimer defects, thus challenging the interpretation put forward in previous work.^7^ In order to further corroborate this hypothesis and to unravel the detailed reaction mechanism, a comprehensive set of DFT and ab initio calculations using the Turbomole^9^ and ORCA^10^ program packages was carried out.

We started with calculations at the M06 level of theory^11^ employing an intact paddle wheel unit and found that CO and O_2_ are not able to adsorb simultaneously on the same Cu^2+^ metal site, but could exchange with one another with a relatively low barrier (less than 0.2 eV). We performed an extensive search for a transition state of CO oxidation with molecular O_2_ (and vice versa) on the pristine paddle wheel. Based on relaxed scans (see Supporting Information for more details) the smallest barrier is estimated to be higher than 2 eV, no barriers with lower energies were found. On the basis of this rather extensive set of calculations we conclude that low‐temperature CO oxidation at the intact Cu^2+^/Cu^2+^ pairs is not possible.

We thus turned our attention to the reduced Cu^+^/Cu^2+^ dimers. First, the extra electron available at the Cu^+^ ion increases the CO binding energy to almost twice the value for the fully oxidized case, Δ*G*=0.38 vs. 0.16 eV (at 100 K). Second, as a result of the additional space (see above), now O_2_ and CO can bind simultaneously on the metal site (see Figure 3). Starting from a O_2_‐Cu^2+^‐Cu^+^‐CO complex (**III**), the reaction path for the overall oxidation of CO contains two transition states, (**TS‐I**) where the first C−O bond forms and (**TS‐II**) that leads to the formation of the second C−O bond with simultaneous cleavage of the O−O bond. We note that **TS‐I** has significant multi‐configurational and multi‐reference character. The calculated value for M06 is over 1.2 eV, while CASSCF^12^ and NEVPT2^13^ that explicitly account for the multi‐reference character calculate **TS‐I** to 0.56 eV (see Supporting Information for more details). This value is in fair agreement with the experimental observation of CO oxidation at temperatures as low as 105 K. **TS‐II** on the other hand, can be successfully treated with single‐reference methods and has a barrier of 0.43 eV (see Supporting Information for more details).

**Figure 3:**
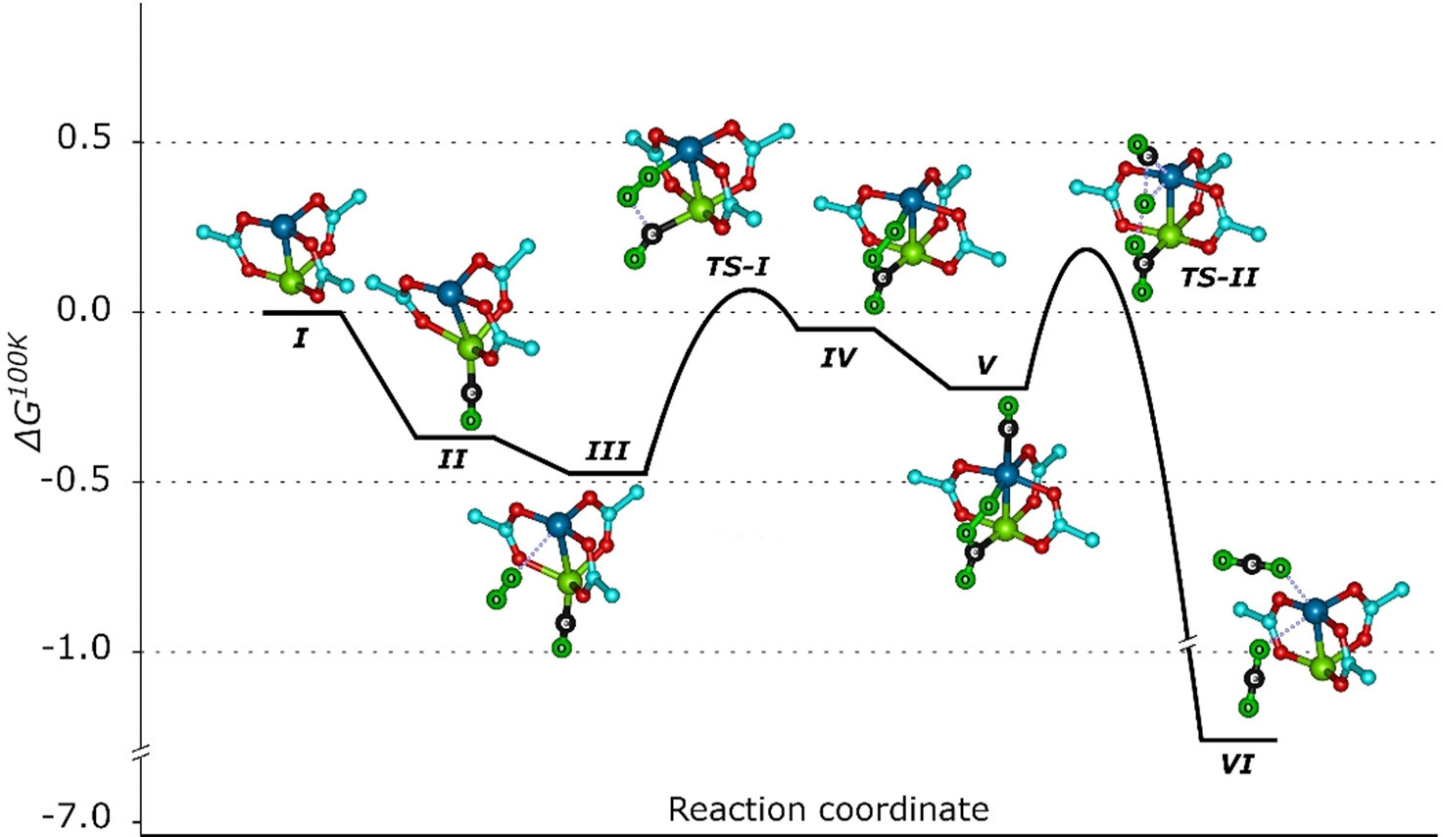
The reaction mechanism (Gibbs free energy diagram at 100 K) of low‐temperature CO oxidation with O_2_ to CO_2_ on DE‐HKUST‐1. All calculations are obtained using the M06 functional, the energy of **TS‐I** has been corrected using NEVPT2 calculations as it has shown to exhibit high multi‐reference character (see Supporting Information for details). All energies and transition states are referenced relative to DE‐HKUST‐1 and CO and O_2_ in the gas phase. All entropy corrections are calculated within the harmonic approximation using M06. C of MOF‐cyan, O of MOF‐red, Cu^2+^‐blue, Cu^+^‐green, C and O atoms of educts/products are shown in black and lime with corresponding captions.

While the activation energies related to this reaction path are fully consistent with the experimental findings, the presence of an uncharged O_2_ species is somewhat surprising (see Figure 4 a,b). In previous reaction schemes for CO oxidation, typically activated oxygen species such as superoxo or peroxo as well as surface lattice oxygen species were considered.^14^ It has been reported that the coadsorption of these activated oxygen species with CO can lead to a frequency shift of the CO stretching vibration due to electronic modifications (see for example, Au/TiO_2_ 15 or Au/ZnO^16^). This was not observed in the present IRRAS data.

**Figure 4:**
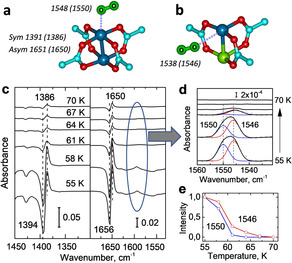
Interaction between dioxygen and HKUST‐1 SURMOFs. DFT‐optimized atomic structures of O_2_ adsorbed to a) Cu^2+^/Cu^2+^ dimers of pristine HKUST‐1 as well as b) to Cu^+^/Cu^2+^ defects of DE‐HKUST‐1, together with the computed O_2_ and carboxylate stretch frequencies. For comparison, the corresponding experimental values are given in the parenthesis. c) IRRAS data in the region of carboxylate vibrations acquired after O_2_ adsorption on DE‐HKUST‐1 SURMOF at 55 K and heating to indicated temperatures. d) Magnification of the O‐O stretching region 1560–1530 cm^−1^, the data were deconvoluted by fitting the individual components. e) Relative intensity evolution of the O‐O vibration at 1550 and 1546 cm^−1^ as a function of temperature. C of MOF‐cyan, O of MOF‐red, Cu^2+^‐blue, Cu^+^‐green, O of O_2_‐lime.

A crucial test for the proposed reaction mechanism in Figure 3 is to demonstrate the presence of the activated O_2_ species adsorbed on Cu CUS. While the O_2_ stretching vibration is IR‐inactive in the gas phase, the weak binding to metal cations results in a small transition dipole moment, so that an observation by IR should, in principle, be possible.^17^ Indeed, after exposing the pristine HKUST‐1 SURMOF to O_2_ at 55 K, a very weak signal at 1550 cm^−1^ (Supporting Information, Figure S4) was clearly observed, slightly red‐shifted with respect to the gas phase value (1556 cm^−1^),^18^ similar to the redshift of 9 cm^−1^ predicted by DFT calculations (Figure 4 a). Note, that also the framework carboxylate‐related vibrations at 1394/1656 cm^−1^ (ν_s_(OCO)/ν_as_(OCO)) showed small red‐shifts to 1386/1650 cm^−1^ (Figure 4 c, Supporting Information, Figure S4) as a result of the dioxygen adsorption, in line with the DFT calculations (redshift of 3 and 5 cm^−1^, respectively, Figure 4 a, see Supporting Information for a detailed discussion).

Similar results were observed after O_2_ adsorption on the DE‐HKUST‐1 SURMOF at 55 K (Figure 4 c). The IRRAS data shown in Figure 4 d allowed identifying two O_2_ species at 1546 and 1550 cm^−1^. The spectra recorded after slight annealing revealed that the binding energies of both O_2_ species are extremely low: the pristine Cu^2+^‐related O_2_ band at 1550 cm^−1^ disappears at about 60 K, while the signal at 1546 cm^−1^ disappears only after heating to 67 K (Figure 4 e). Based on DFT calculations, the latter one is assigned to activated, uncharged dioxygen species adsorbed to Cu^2+^ CUS of the reduced Cu^+^/Cu^2+^ dimers (Figure 4 b). Again, the computed frequency (10 cm^−1^ red‐shift in comparison to O_2_ on pristine paddle wheel) and binding energy (0.1 eV) are in good agreement with the experimental results that show the binding only occurs at low temperatures.

After providing strong support for the reaction pathway described in Figure 3 by demonstrating the presence of the uncharged O_2_ as an important intermediate, we come to the experimental observation that upon exposure to O_2_, for the DE‐HKUST‐1 only the CO‐Cu^2+^ band decreases in intensity, while the intensity of the band assigned to the CO‐Cu^+^, which should be consumed during the reaction, stays virtually constant (Figure 2 b). Considering that the binding energy is larger (by 0.2 eV, according to our DFT calculations) in the latter case, this observation can be explained by a quick replenishing of the consumed CO‐Cu^+^ species by direct transfer from adjacent Cu^2+^ sites, where CO is more weakly bound. Indeed, a careful analysis of the coverage‐dependent IRRAS data (Supporting Information, Figure S5) revealed that the activation energy to transfer CO from Cu^2+^ to Cu^+^ sites must be lower than 0.20 eV, assuming a diffusion prefactor of 2.5×10^−8^ cm^2^ s^−1^.^19^ Similarly, our DFT calculations result in a binding energy of CO to the ideal pristine paddle wheel of about 0.4 eV that, taking entropy corrections at 100 K into account, leads to a desorption barrier of 0.16 eV.

In summary, on the basis of the experimental observations and the theoretical results, we can provide a fully consistent reaction scheme for low‐temperature CO oxidation occurring in defect‐engineered MOFs of type HKUST‐I, with the reaction proceeding along the potential path depicted in Figure 3. We find that creating defects in HKUST‐1 not only yields higher binding energy adsorption sites for CO, but also additional space to allow for the simultaneous binding of CO and dioxygen on Cu^+^/Cu^2+^ single dimers in a synergistic fashion. The unexpected finding of an uncharged dioxygen intermediate is corroborated by high‐sensitivity IRRAS data. These results demonstrate that a detailed analysis of reactions in structurally well‐defined metal‐organic frameworks with their interplay of electronic and steric effects is possible, thus opening the path for a systematic tuning of MOF catalytic properties. Particularly interesting in this context will be mixed‐metal systems, for which high catalytic activity has been observed.^20^

## Conflict of interest

The authors declare no conflict of interest.

## Supporting information

As a service to our authors and readers, this journal provides supporting information supplied by the authors. Such materials are peer reviewed and may be re‐organized for online delivery, but are not copy‐edited or typeset. Technical support issues arising from supporting information (other than missing files) should be addressed to the authors.

Supplementary
