# Supplementary material for: Interplay of Electronic and Steric Effects to Yield Low‐Temperature CO Oxidation at Metal Single Sites in Defect‐Engineered HKUST‐1
Source: Angew Chem Int Ed Engl. 2020 Apr 17;59(26):10514–8. doi: 10.1002/anie.202000385 (PMC7318571; doi:10.1002/anie.202000385)
Supplement: Supplementary file 1 — Supplementary [file ANIE-59-10514-s001.pdf]

Angewandte Chemie
Eine Zeitschrift der Gesellschaft Deutscher Chemiker

Supporting Information

Interplay of Electronic and Steric Effects to Yield Low-Temperature CO Oxidation at Metal Single Sites in Defect-Engineered HKUST-1

Weijia Wang[+], Dmitry I. Sharapa[+], Abhinav Chandresh, Alexei Nefedov, Stefan Heißler, Lars Heinke, Felix Studt, Yuemin Wang,* and Christof Wöll**

anie_202000385_sm_miscellaneous_information.pdf

Materials and Methods

(1) Preparation of HKUST-1 SURMOFs

A layer by layer (LbL) approach was used for preparation of monolithic HKUST-1 thin films (surface-mounted MOFs, SURMOFs). In this approach, the HKUST-1 thin films were grown on a gold coated silicon wafer which is orientated in (100) direction. Gold was coated using indirect thermal evaporation method. The thickness of the gold layer is 100 nm, a 5nm layer of titanium (grade 5N) was used as adhesive. The substrates were first functionalized with self-assembled monolayers of 20 μM MHDA (16-Mercaptohexadecanoic acid) solution (acetic acid/ethanol v/v=1/9) at room temperature for 3 days, followed by rinsing with pure ethanol and N2 drying. After drying, substrates were immediately submerged into metal solution. The functionalized substrates were dipped into ethanolic 1 mM copper(II) acetate and ethanolic 0.2 mM BTC (benzene-1,3,5- tricarboxylic acid) solutions for 5 and 15 minutes alternatively. Between these dipping steps, 2 rinsing steps with pure ethanol for 1 minute each were also included in the process to remove the extra material left from the previous solution. The total number of cycles used were 50.

(2) IRRAS and XPS experiments.

The in situ IRRAS experiments were conducted in an advanced multitechnique UHV apparatus, which combines X-ray photoelectron spectroscopy (XPS), ultraviolet photoelectron spectroscopy (UPS), low-energy electron diffraction (LEED) and UHV Fourier-transform infrared spectroscopy (UHV-FTIRS). The base pressure of the UHV chamber was 8.0×10^{-11} mbar. This apparatus has been optimized for sensitivity and allows to reliably detect absorbance as low as 1×10^{-5}. A comprehensive introduction of the experimental setup can be found elsewhere (*1*).

The pristine and defect-engineered HKUST-1 (DE-HKUST-1) SURMOFs were characterized by IRRAS using CO as a probe molecule at low temperatures (55-85 K, cooling with liquid helium). The DE-HKUST-1 samples were prepared through heating the pristine one to higher temperatures in UHV in a controlled fashion. The oxidation states of various MOF samples were further monitored by grazing incidence XPS equipped with a VG Scienta R4000 electron energy analyzer. The catalytic activity of pristine and DE-HKUST-1 SURMOFs towards low temperature CO oxidation reaction was measured at 105 K. Dosing of CO and O_2 was performed through a capillary tube ending in front of the sample. The sample temperature was monitored by a K-type thermocouple that was placed on the edge of the sample holder. Prior to each exposure, a spectrum of the clean SURMOF sample was recorded as a background reference. All IR data shown here are difference spectra obtained by subtracting the reference. The IRRAS data were accumulated by recording typically 1024 scans with a resolution of 4 cm^{-1} at a grazing incidence angle of 10°.

(3) Computational details

All DFT results were obtained with Turbomole package (*2-8*). All structures were fully optimized using the M06 functional (*9*) and def2-TZVP basis set (*10,11*). The convergence criteria were 10^{-6} Hartree for energies and 10^{-3} atomic units for maximum norm of cartesian gradient. A subset of the most complex structures was calculated, for comparative purposes also with B3LYP (*12-15*) /def2-TZVP and B2PLYP (*16*) /def2-TZVP.

Vibrational modes were calculated analytically as implemented in aoforce module of Turbomole. The entropic contribution to the free energy was obtained from the calculated vibrations. For comparison with experimentally measured frequencies and corresponding shifts, the computed IR frequencies are normalized in following way: O_2 and CO stretching frequencies are normalized to the vibrations of the corresponding molecules in vacuum. The symmetric and asymmetric carboxylate vibrations are normalized to the corresponding vibrations of carboxylate group at pristine paddle-wheel structure. Thus, different scaling coefficients were used for each kind of vibration (see Table S1). Note, that these coefficients are in range with other scaling coefficients published in literature for M06/def2-TZVP (0.91-0.97) (*17,18*). Note that the tables S6-S27 give the nonscaled/nonnormalized frequencies as calculated with M06.

Fractional occupation density (FOD) diagnostics (*19,20*) were performed with ORCA 4.0.0 (*21-22*) and revealed high multi configurational multi reference character of **TS-I** (see Figure S5). **TS-I** has therefore been reoptimized using CASSCF (*23*) with single point calculations using NEVPT2 (*24*) on the optimized structure as implemented in the ORCA 4.0.0 package. (*25*) In all cases the basis set was converged.

CAS optimizations of **TS-I** can be performed on active space of 3x3, after localization these “active” orbitals can be described as “2 orbitals on copper and 1 orbital on O_2”. However, in **III** two of three orbitals with unpaired electrons located on O_2 and one on copper to which CO is bounded. Thus, for comparison of these structures, active space of 5 electrons on 4 orbitals (one on each Cu and 2 on O_2) has to be used (set of these orbitals shown on the last page of this SI).

Using the CASSCF(5x4) and NEVPT2(5x4) calculations on **TS-I** and **III** we obtain a barrier of 0.33 and 0.36 eV, respectively as opposed to 1.03 eV calculated with M06 (See Table S4). We thus corrected the energy of **TS-I** using NEVPT2 as a reference. We confirmed the validity of this approach by calculating the energy difference between **III** and **IV** with CASSCF(5x4)/def2-TZVP. These calculations yield and energy difference of 0.33 eV which compares to 0.35 eV using M06.

All structures were visualized with Chemcraft.(*26*) Hydrogen atoms in the manuscript were omitted for clarity. Copper of different spin states has different coloring for clarity.

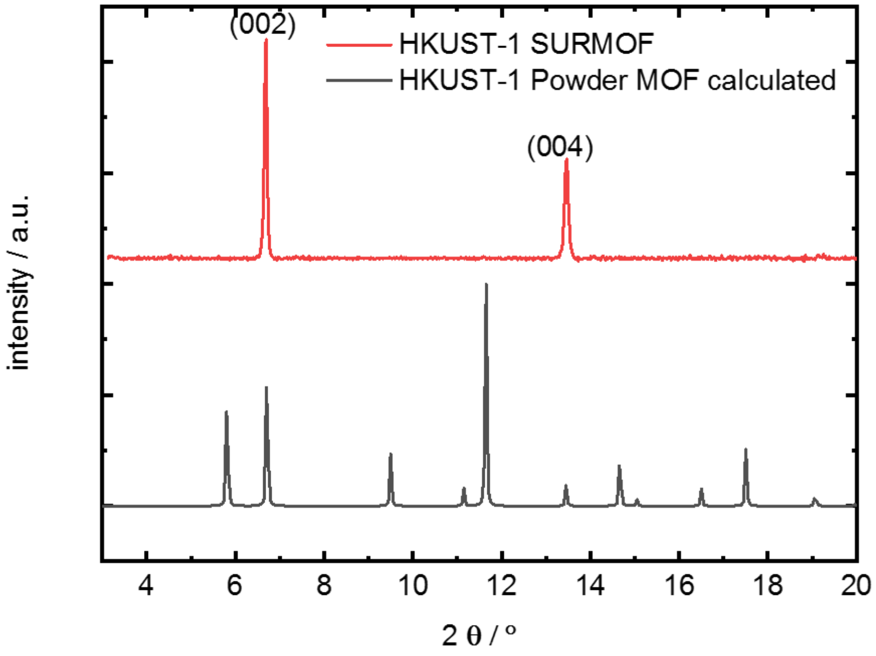

Figure S1. X-ray diffractograms of HKUST-1 SURMOF measured in the out-of-plane geometry. For comparison, the calculated XRD patterns for HKUST-1 in the form of powders are also presented.

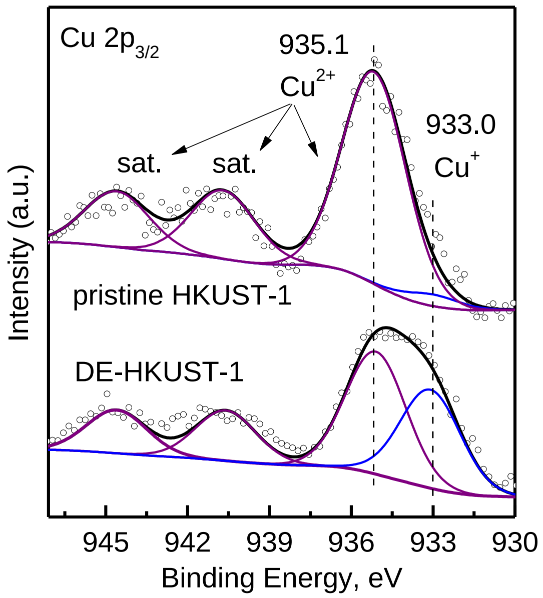

Figure S2. Pristine and DE-HKUST-1 SURMOFs characterized by XPS. Cu $2p_{3/2}$ XPS spectra of the pristine (a) and DE-HKUST-1 SURMOF (b). The DE-HKUST-1 SURMOF with the Cu^{+} concentration of 30 % was obtained by heating to 430 K for 30 min.

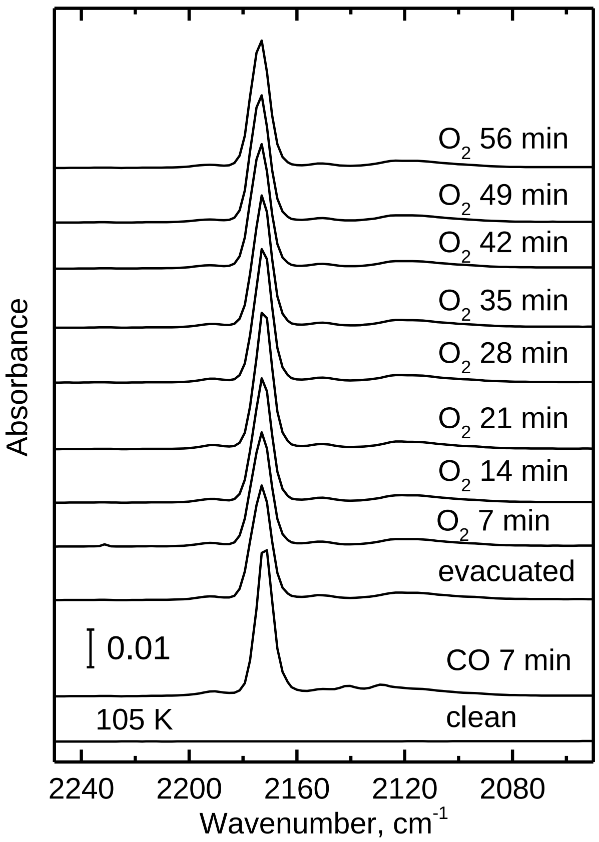

Figure S3. **Evaluation of the chemical reactivity of defect-free HKUST-1 SURMOFs**. IRRAS data recorded during exposing the pristine HKUST-1 SURMOF first to CO (1×10^{-5} mbar) and then to O_2 (1×10^{-5} mbar) for different times at 105 K.

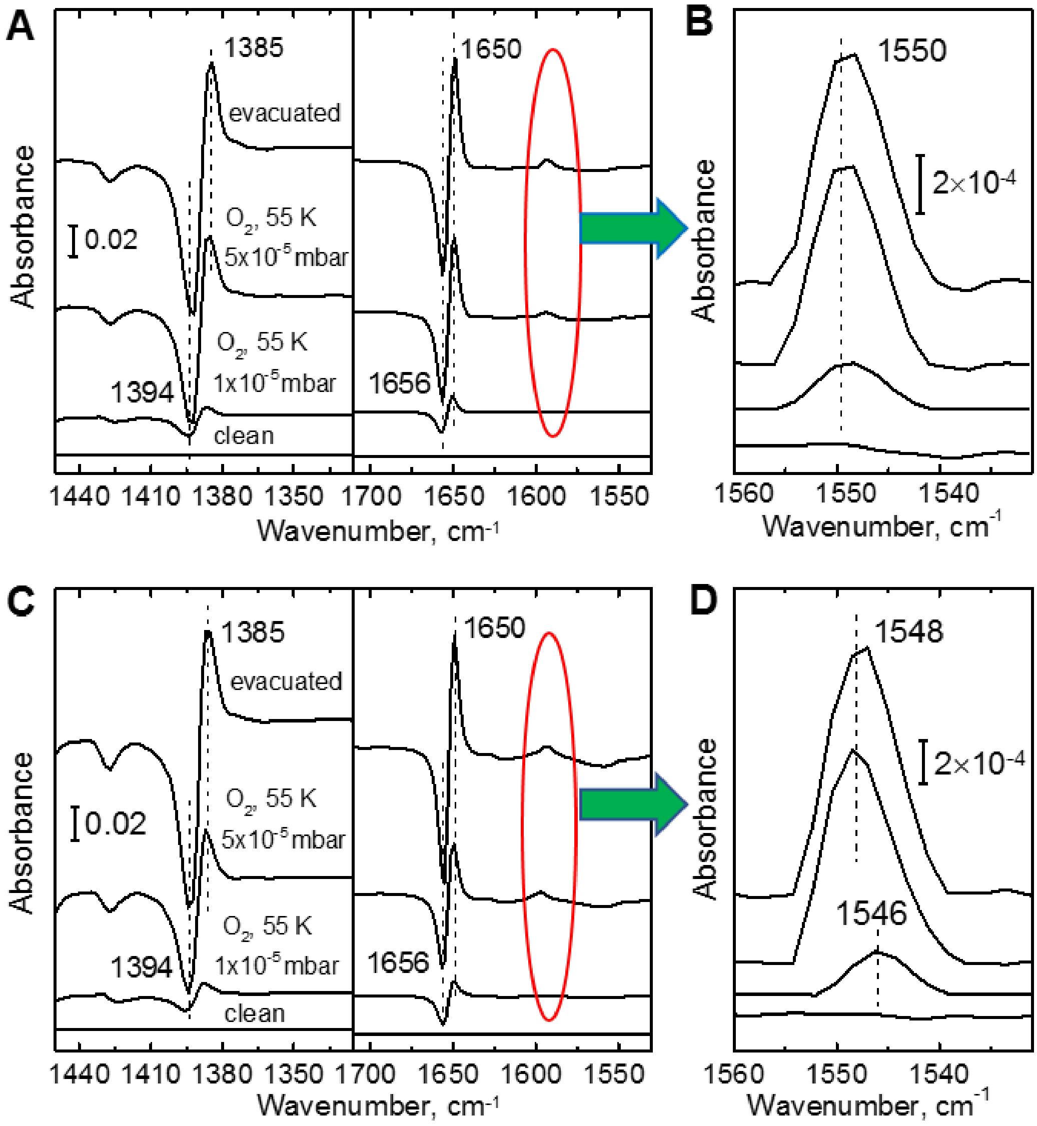

Figure S4. **Interaction between dioxygen and HKUST-1 SURMOFs**. (**A**) RRAS data in the region of carboxylate vibrations acquired after O_2 adsorption on pristine HKUST-1 SURMOF at 55 K for different exposures. (**B**) Magnification of the O–O stretching region 1560-1530 cm^{-1} in A). (**C**) IRRAS data in the region of carboxylate vibrations acquired after O_2 adsorption on DE-HKUST-1 SURMOF at 55 K for different exposures. (**D**) Magnification of the O–O stretching region 1560-1530 cm^{-1} in C). The DE-HKUST-1 SURMOF with the Cu^+ concentration of 30 % was obtained by heating to 430 K for 30 min.

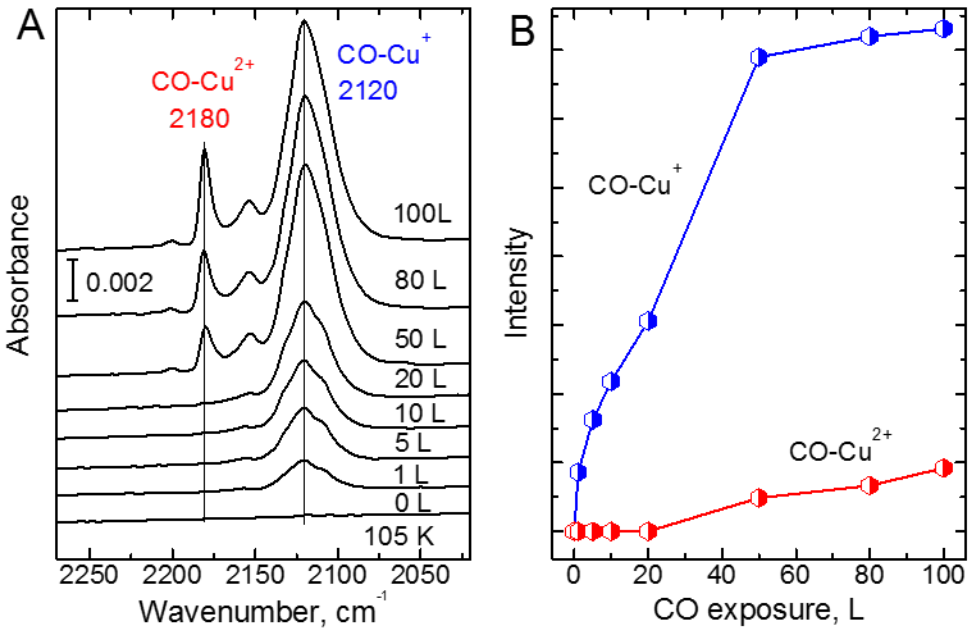

Figure S5. **Interaction between CO and DE-HKUST-1 SURMOFs**. (**A**) IRRA spectra acquired after CO adsorption on the DE-HKUST-1 SURMOF for different exposures at 105 K; B) Integrated intensity evolution of the two spectral components in A). The DE-HKUST-1 SURMOF with the Cu^+ concentration of about 40 % was obtained by heating the pristine HKUST-1 to 450 K for 30 min.

The activation energy E_a for CO hopping from Cu^{2+} to Cu^+ CUS was estimated from the temperature-dependence of the diffusivity D, given by

$D = D_0 \exp(-E_a/kT)$

where D_0 is the diffusion prefactor and k is the Boltzmann constant.

For one-dimension hopping, $D = \frac{1}{2} \Gamma_s x^2$ (*27*)

and $P_0(t) = \exp(-\Gamma_s t)$

where Γ_s is the hopping rate; x is the hopping distance (for HKUST-1, the Cu^{2+}-Cu^+ distance is 2.58 Å (*28*)); $P_0(t)$ is the probability that adsorbates do not move in the time interval t.

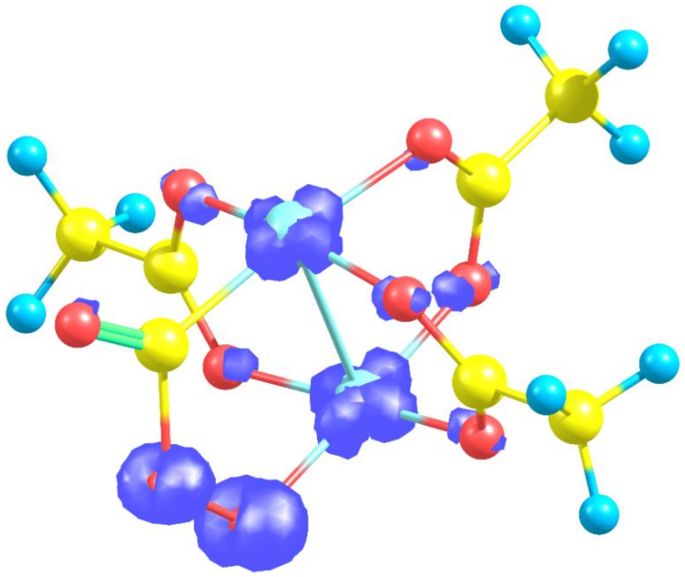

Figure S6. FOD-diagnostic TS-I, isosurface 0.02.

References

1. IR spectroscopic investigations of chemical and photochemical reactions on metal oxides: bridging the materials gap. Y. Wang, C. Woll, *Chem. Soc. Rev.* **46**, 1875-1932 (2017). doi: 10.1039/C6CS00914J

2. TURBOMOLE V7.0 2015, a development of University of Karlsruhe and Forschungszentrum Karlsruhe GmbH, 1989-2007, TURBOMOLE GmbH, since 2007; available from http://www.turbomole.com.

3. Electronic Structure Calculations on Workstation Computers: The Program System TURBOMOLE. R. Ahlrichs, M. Bär, M. Häser, H. Horn and C. Kölmel; *Chem. Phys. Letters* **162**, 165 (1989). doi: 10.1016/0009-2614(89)85118-8

4. Efficient Molecular Numerical Integration Schemes. O. Treutler and R. Ahlrichs; *J. Chem. Phys.* **102**, 346 (1995). doi: 10.1063/1.469408

5. Auxiliary basis sets for main row atoms and transition metals and their use to approximate Coulomb potentials. K. Eichkorn, F. Weigend, O. Treutler and R. Ahlrichs; *Theor. Chem. Acc.* **97**, 119 (1997). doi: 10.1007/s002140050244

6. Auxiliary Basis Sets to Approximate Coulomb Potentials. K. Eichkorn, O. Treutler, H. Öhm, M. Häser and R. Ahlrichs; *Chem. Phys. Letters* **242**, 652 (1995). doi: 10.1016/0009-2614(95)00621-A

7. Fast evaluation of the Coulomb potential for electron densities using multipole accelerated resolution of identity approximation. M. Sierka, A. Hogekamp and R. Ahlrichs; *J. Chem. Phys.* **118**, 9136, (2003). doi: 10.1063/1.1567253

8. Nuclear second analytical derivative calculations using auxiliary basis set expansion. P. Deglmann, K. May, F. Furche and R. Ahlrichs; *Chem. Phys. Letters* **384**, 103. (2004) doi: 10.1016/j.cplett.2003.11.080

9. The M06 suite of density functionals for main group thermochemistry, thermochemical kinetics, noncovalent interactions, excited states, and transition elements: two new functionals and systematic testing of four M06-class functionals and 12 other functionals. Y. Zhao, D.G. Truhlar, *Theor Chem Account* 120: 215. (2008) doi: 10.1007/s00214-007-0310-x

10. Balanced basis sets of split valence, triple zeta valence and quadruple zeta valence quality for H to Rn: Design and assessment of accuracy. F. Weigend and R. Ahlrichs, *Phys. Chem. Chem. Phys.*, **7** (2005) 3297-305. doi: 10.1039/B508541A

11. Accurate Coulomb-fitting basis sets for H to Rn. F. Weigend, *Phys. Chem. Chem. Phys.*, **8** (2006) 1057-65. doi: 10.1039/B515623H

12. Density-functional thermochemistry. III. The role of exact exchange A.D. Becke, *J.Chem.Phys.* **98** (1993) 5648-5652 10.1063/1.464913

13. Development of the Colle-Salvetti correlation-energy formula into a functional of the electron density C. Lee, W. Yang, R.G. Parr, *Phys. Rev. B* **37** (1988) 785-789 doi: 10.1103/PhysRevB.37.785

14. Accurate spin-dependent electron liquid correlation energies for local spin density calculations: a critical analysis. S.H. Vosko, L. Wilk, M. Nusair, *Can. J. Phys.* **58** (1980) 1200-1211 doi: 10.1139/p80-159

15. Ab Initio Calculation of Vibrational Absorption and Circular Dichroism Spectra Using Density Functional Force Fields P.J. Stephens, F.J. Devlin, C.F. Chabalowski, M.J. Frisch, *J.Phys.Chem.* **98** (1994) 11623-11627 doi: 10.1021/j100096a001

16. Semiempirical hybrid density functional with perturbative second-order correlation. S. Grimme, *J. Chem. Phys.*, **124** (2006) 034108. doi: 10.1063/1.2148954

17. Database of Frequency Scale Factors for Electronic Model Chemistries (maintained by J. Zheng, I. M. Alecu, B. J. Lynch, Y. Zhao, and D. G. Truhlar), available from https://comp.chem.umn.edu/freqscale/version3b1.htm or https://comp.chem.umn.edu/freqscale/190107_Database_of_Freq_Scale_Factors_v4.pdf

18. Frequency and Zero-Point Vibrational Energy Scale Factors for Double-Hybrid Density Functionals (and Other Selected Methods): Can Anharmonic Force Fields Be Avoided? Manoj K. Kesharwani, B. Brauer, J. M. L. Martin. *J. Phys. Chem. A* **119**, 9, 1701-1714, (2015), 10.1021/jp508422u

19. A Practicable Real-Space Measure and Visualization of Static Electron-Correlation Effects. S. Grimme, A. Hansen; *Angew. Chem. Int. Ed.*, **54**: 12308-12313. (2015), doi:10.1002/anie.201501887

20. The Fractional Occupation Number Weighted Density as a Versatile Analysis Tool for Molecules with a Complicated Electronic Structure. C. A. Bauer, A. Hansen, S. Grimme; *Chem. Eur. J.*, *23*, 6150. (2017). doi: 10.1002/chem.201604682

21. The ORCA program system. F. Neese; *WIREs Comput Mol Sci*, **2**,(1), 73–78. (2012) doi:10.1002/wcms.81

22. Software update: the ORCA program system, version 4.0. F. Neese; *WIREs Comput Mol Sci*, e1327. (2017) doi: 10.1002/wcms.1327

23. The CASSCF method: A perspective and commentary. Olsen, J. *Int. J. Quantum Chem.*, 111: 3267-3272. (2011), doi:10.1002/qua.23107

24. Assessment of n-Electron Valence State Perturbation Theory for Vertical Excitation Energies. Schapiro, I.; Sivalingam, K.; F. Neese, *J. Chem. Theory Comput.*, 9(8), 3567. (2013) doi: 10.1021/ct400136y

25. CASSCF Calculations in ORCA (4.2): A tutorial Introduction. D. Aravena, M. Atanasov, V. G. Chilkuri, Y. Guo, J. Jung, D. Maganas, B. Mondal, I. Schapiro, K. Sivalingam, S. Ye and F. Neese a supplement of the ORCA 4.0.0 manual, available from https://orcaforum.kofo.mpg.de/

26. G.A. Zhurko, ChemCraft software, version 1.8. (http://www.chemcraftprog.com)

27. Microscopic molecular diffusion enhanced by adsorbate interactions. B. G. Briner, M. Doering, H. P. Rust, A. M. Bradshaw, *Science* **278**, 257-260. (1997), doi: 10.1126/science.278.5336.257

28. Insights into Adsorption of NH3 on HKUST-1 Metal-Organic Framework: A Multitechnique Approach. E. Borfecchia, S. Maurelli, D. Gianolio, E. Groppo, M. Chiesa, F. Bonino, C. Lamberti, *J. Phys. Chem. C* **116**, 19839-19850. (2012), doi: 10.1021/jp305756k

Table S1. Normalisation factors for IR-frequencies

	Experiment	M06	Scaling factor
**O_2**	1556	1711	0.9094
**CO**	2143	2236	0.9584
**Symmetric carboxylate of PW**	1394	1488	0.9368
**Symmetric carboxylate of PW**	1656	1715	0.9656

Table S2. Binding energies of O_2 and CO to defected PW and their structures:

Name	Total energy (a.u)	Binding energy, eV
**Defect (№ I on Figure 3)**	-3966.45243	
**CO**	-113.30141	
**O_2**	-150.31419	
**Cu(I)_CO (№ II on Figure 3)**	-4079.77578	-0.60
**Cu(II)_CO**	-4079.76564	-0.32
**Cu(I)_OO_Doublet**	-4116.76628	0.01
**Cu(II)_OO_Doublet**	-4116.76909	-0.07
**Cu(I)_OO_Quartet**	-4116.76699	-0.01
**Cu(II)_OO_Quartet**	-4116.76715	-0.01

Table S3. Enthalpies, entropies and Gibbs free energies of structures shown on Figure 3

	Enthalpy (hartree)		Entropy	Gibbs Free Energy (hartree)	
	Total	Relative	kcal/mol	Total	Relative
**Defect doublet (I)**	-3966.4524	0.00	147	-3966.4758	0.00
**Defect-CO doublet (II)**	-4079.7758	-0.60	145	-4079.7989	-0.39
**O_2-Defect-CO quartet (III)**	-4230.0940	-0.71	193	-4230.1247	-0.49
**TS-I doublet**	-4230.0563	0.32	143	-4230.0791	0.75
**Cycle doublet(IV)**	-4230.0810	-0.35	172	-4230.1084	-0.05
**Cycle-CO doublet (V)**	-4343.3960	-0.73	175	-4343.4239	-0.23
**TS-II doublet**	-4343.3803	-0.30	159	-4343.4057	0.27
**Product doublet (VI)**	-4343.6310	-7.12	191	-4343.6614	-6.69
**O_2 triplet**	-150.3142		49	-150.3220	
**CO singlet**	-113.3013		47	-113.3089	
**CO_2 singlet**	-188.5819		52	-188.5903	

Table S4. Process of CO-O_2 exchange on pristine PW (paddle wheel)

	Total energy	kcal/mol	Entropy, cal/mol/K	deltaG	Free energy
**HKUST-CO…O2**	-4458.558367	-9.34	164.123	-4458.584522	-3.65
**TS (exchange)**	-4458.551292	-4.90	167.023	-4458.577909	0.49
**HKUST-O2….CO**	-4458.552052	-5.38	169.612	-4458.579082	-0.24

All calculations performed with M06/def2-TZVP, triplet state, hydrogens frozen in same position (optimal for pristine PW) to avoid systematic problems with rotation of methyl groups.

Table S5 Total and relative energies of first reaction barrier obtained by different methods (no entropy).

	M06	CASSCF (3x3)	NEVPT2 (3x3)	CASSCF(5x4)	CASSCF (5x4)	NEVPT2 (5x4)
	def2-TZVP	def2-SVP	def2-SVP	def2-SVP	def2-TZVP	def2-TZVP
**III**	-4230.0940	-4220.9133	-4224.0910	-4220.9027	-4222.4774	-4226.8050
**TS-I**	-4230.0563	-4220.8901	-4224.0669	-4220.8901	-4222.4644	-4226.7929
eV	1.03	0.63	0.65	0.34	0.36	0.33

Table S6. Charges and spin densities of **TS-I**:

CAS charges and spin densities of **TS-I**:		Mulliken charges and spin densities of **TS-I** from DFT:	
Loewdin	Mulliken	M06/def2-TZVP	B3LYP/def2-TZVP
Cu 0.409301 0.317357	Cu 0.729544 0.321733	Cu 0.101434 0.090156	Cu 0.142033 0.088662
Cu 0.539554 0.754916	Cu 0.935802 0.762604	Cu 0.293720 0.483825	Cu 0.339690 0.504424
O 0.024769 0.014039	O -0.603354 0.012219	O -0.393570 0.063107	O -0.352090 0.057610
C -0.483480 0.003336	C 0.711490 0.000688	C 0.599615 -0.004953	C 0.498414 -0.001531
O 0.055025 0.003483	O -0.619628 0.001935	O -0.431257 0.009305	O -0.381371 0.004249
O 0.054703 0.003473	O -0.621840 0.001922	O -0.440193 0.014066	O -0.388078 0.012845
O 0.037876 0.005103	O -0.599642 0.005099	O -0.414198 0.018181	O -0.364086 0.016594
C -0.483829 0.003336	C 0.713245 0.000681	C 0.608797 -0.002688	C 0.511244 -0.000713
O 0.024912 0.014044	O -0.603211 0.012217	O -0.394964 0.070386	O -0.355761 0.066267
O 0.049759 0.009163	O -0.615646 0.006477	O -0.420355 0.097555	O -0.372845 0.093378
C -0.486137 0.002155	C 0.724976 0.000879	C 0.632274 -0.010456	C 0.503823 -0.004980
O -0.076822 0.563084	O -0.217298 0.581409	O -0.085911 0.145250	O -0.122200 0.147960
O -0.116195 0.285589	O -0.259951 0.278751	O -0.078957 0.020204	O -0.088176 0.017269
C -0.257249 0.015353	C 0.317305 0.008067	C 0.380645 -0.002412	C 0.288544 -0.010168
O 0.198138 0.003445	O -0.228323 0.002758	O -0.100511 -0.012906	O -0.047960 -0.013873
C -0.217904 0.000589	C -0.324213 0.000860	C -0.295786 0.004832	C -0.333896 0.004192
H 0.125995 0.000045	H 0.133177 0.000030	H 0.116875 -0.000118	H 0.134136 0.000532
H 0.125809 0.000065	H 0.132625 0.000066	H 0.111272 0.001696	H 0.134517 0.001499
H 0.137554 0.000027	H 0.134980 0.000005	H 0.119069 0.000208	H 0.132859 -0.000318
C -0.219218 0.000557	C -0.308501 0.000506	C -0.287022 0.008701	C -0.333915 0.010180
H 0.126107 0.000035	H 0.132221 0.000039	H 0.107267 0.000104	H 0.130484 0.000194
H 0.125502 0.000061	H 0.134133 0.000073	H 0.108891 0.000977	H 0.125851 -0.000120
H 0.134288 0.000018	H 0.125668 0.000026	H 0.109209 -0.000239	H 0.130679 0.001068
C -0.217829 0.000590	C -0.324394 0.000855	C -0.292005 0.004094	C -0.334175 0.003741
H 0.126109 0.000045	H 0.133147 0.000030	H 0.115492 -0.000083	H 0.135117 0.001039
H 0.137474 0.000027	H 0.134900 0.000005	H 0.117211 0.000016	H 0.133717 0.000336
H 0.125789 0.000065	H 0.132789 0.000067	H 0.112958 0.001195	H 0.133448 -0.000337

All charges were calculated on the structures optimized on the same level of theory (i.e. CASSCF, M06, B3LYP correspondingly)

Table S7. Ideal (pristine) paddle-wheel (singlet biradical, localized on Cu atoms)

30				Assymetic OCO vibration (1715 cm^{-1})	Symmetric OCO vibration (1488 cm^{-1})	Symmetric OCO vibration (1488 cm^{-1})
O	-1.454661000	-1.296200000	1.126128000	-0.04 -0.05 -0.14	0.01 0.01 0.02	-0.05 -0.06 -0.09
C	-1.798594000	-1.763696000	0.011352000	*-0.02 0.03 0.38*	*-0.04 -0.04 -0.00*	*0.21 0.20 -0.03*
O	-1.325822000	-1.434379000	-1.106727000	0.05 0.04 -0.13	0.01 0.01 -0.02	-0.07 -0.05 0.11
Cu	0.005379000	0.000901000	1.232686000	-0.00 -0.00 0.00	0.00 -0.00 0.00	0.00 0.00 -0.00
Cu	0.001348000	-0.005122000	-1.231689000	0.00 -0.00 0.00	0.00 -0.00 -0.00	0.00 0.00 0.00
O	-1.428601000	1.323490000	-1.119370000	0.04 -0.05 -0.13	-0.05 0.06 0.10	-0.01 0.01 0.02
C	-1.762223000	1.796109000	-0.003289000	*0.02 0.02 0.38*	*0.22 -0.20 -0.00*	*0.05 -0.05 0.01*
O	-1.290367000	1.460372000	1.112976000	-0.05 0.04 -0.13	-0.07 0.05 -0.10	-0.01 0.01 -0.03
O	1.300235000	-1.460064000	1.121269000	0.05 -0.04 -0.14	-0.06 0.05 0.09	0.00 0.00 -0.00
O	1.465326000	1.294613000	1.109560000	0.04 0.05 -0.13	0.00 0.00 -0.01	-0.06 -0.07 0.11
C	1.767675000	-1.800582000	0.005128000	*-0.02 -0.02 0.38*	*0.21 -0.20 0.02*	*-0.00 -0.00 0.01*
O	1.431892000	-1.330683000	-1.111970000	-0.04 0.05 -0.13	-0.06 0.07 -0.11	0.00 0.00 -0.00
O	1.328410000	1.426971000	-1.123176000	-0.05 -0.04 -0.14	0.01 0.00 0.01	-0.07 -0.06 -0.10
C	1.799992000	1.764516000	-0.008000000	*0.02 -0.02 0.38*	*-0.01 -0.01 -0.00*	*0.21 0.24 -0.01*
C	2.837437000	-2.850084000	-0.003048000	0.00 0.00 -0.02	-0.06 0.09 -0.01	-0.00 -0.00 0.00
H	2.901365000	-3.350467000	0.960183000	-0.15 0.10 0.05	0.06 -0.29 -0.22	-0.00 0.01 0.01
H	2.647832000	-3.568774000	-0.799289000	0.14 -0.04 -0.01	-0.02 -0.26 0.30	0.01 0.01 -0.01
H	3.793172000	-2.368991000	-0.218883000	0.01 -0.05 -0.07	-0.02 0.05 0.12	0.00 -0.01 -0.01
C	2.839860000	2.843593000	-0.001758000	-0.00 0.00 -0.02	0.01 0.00 -0.00	-0.11 -0.07 0.01
H	3.319536000	2.926645000	-0.974384000	0.08 0.15 0.04	-0.01 -0.01 -0.01	0.39 0.00 0.26
H	2.348783000	3.791767000	0.226243000	-0.03 -0.00 -0.07	0.00 -0.00 0.01	-0.08 -0.04 -0.06
H	3.574471000	2.656206000	0.779507000	-0.05 -0.15 0.00	-0.01 0.00 0.02	0.32 0.02 -0.37
C	-2.840877000	2.836426000	-0.000024000	-0.00 -0.00 -0.02	-0.06 0.10 -0.00	-0.01 0.02 -0.00
H	-2.831631000	3.402365000	-0.929368000	-0.15 0.07 0.03	-0.01 -0.35 -0.28	-0.01 -0.07 -0.06
H	-3.804862000	2.329431000	0.078706000	-0.00 -0.00 -0.07	-0.05 0.07 0.00	-0.01 0.01 -0.01
H	-2.735288000	3.495757000	0.859111000	0.15 -0.06 0.02	0.02 -0.32 0.32	0.02 -0.07 0.07
C	-2.875188000	-2.806265000	-0.000840000	-0.00 -0.00 -0.02	0.01 0.02 -0.00	-0.07 -0.08 0.02
H	-3.174543000	-3.074377000	1.009255000	0.13 0.12 0.06	-0.02 -0.05 -0.03	0.13 0.20 0.16
H	-3.734851000	-2.422712000	-0.551973000	-0.02 -0.09 -0.05	-0.01 0.01 0.03	0.08 -0.04 -0.20
H	-2.518866000	-3.687496000	-0.534657000	-0.11 -0.03 -0.04	0.00 -0.03 0.06	0.00 0.12 -0.26

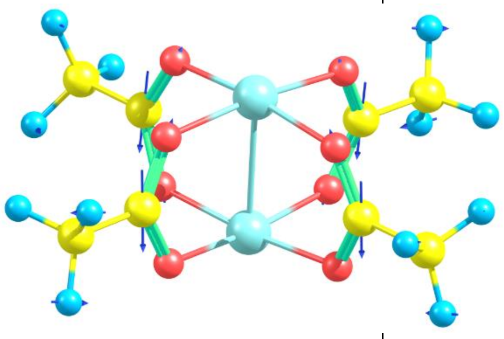

Table S8. Ideal-O_2 (triplet, unpaired electrons are on oxygen molecule)

32				O-O stretching (1702 cm^{-1})	Assymetic OCO vibration (1711 cm^{-1})
O	0.660233000	-1.485971000	-1.753853000	-0.00 0.00 -0.00	-0.09 0.04 -0.11
C	1.466100000	-1.835569000	-0.854424000	0.01 -0.00 0.01	*0.14 0.02 0.35*
O	1.530632000	-1.361984000	0.307545000	-0.00 -0.00 -0.00	-0.01 -0.05 -0.14
Cu	-0.570534000	-0.001656000	-1.439560000	0.00 -0.00 0.00	0.00 0.00 0.00
Cu	0.241526000	-0.004028000	0.897594000	-0.00 -0.00 0.00	0.00 0.00 0.00
O	1.485989000	1.402039000	0.329850000	0.00 0.00 -0.00	0.01 0.04 -0.15
C	1.565733000	1.727729000	-0.881523000	0.01 0.00 0.01	*0.11 0.02 0.36*
O	0.869356000	1.266414000	-1.820480000	-0.00 -0.00 -0.00	-0.08 -0.05 -0.11
O	-1.936107000	-1.270693000	-0.845215000	-0.00 0.00 -0.01	-0.01 0.05 -0.14
O	-1.730663000	1.481360000	-0.917135000	0.00 -0.00 -0.01	0.00 -0.04 -0.14
C	-1.899353000	-1.729609000	0.323780000	0.01 -0.00 0.02	*0.14 -0.03 0.35*
O	-1.078212000	-1.410665000	1.220862000	-0.01 -0.00 -0.01	-0.09 -0.04 -0.11
O	-1.127693000	1.354757000	1.238040000	-0.01 0.00 -0.01	-0.08 0.05 -0.12
C	-1.801234000	1.828584000	0.289256000	0.01 -0.00 0.03	0.11 -0.02 0.36
O	1.327589000	-0.001376000	3.145702000	*-0.70 -0.01 -0.02*	0.02 0.00 0.00
O	2.521059000	0.009142000	3.174746000	*0.71 0.01 0.02*	-0.02 -0.00 -0.00
C	-2.937359000	-2.744316000	0.700207000	-0.00 0.00 -0.00	-0.01 0.00 -0.02
H	-3.496416000	-3.076363000	-0.170993000	-0.01 -0.01 0.01	-0.11 -0.10 0.10
H	-2.460704000	-3.590731000	1.193808000	0.01 0.00 -0.00	0.11 0.03 -0.07
H	-3.621589000	-2.293599000	1.420932000	-0.00 0.01 -0.00	-0.01 0.08 -0.06
C	-2.803482000	2.892548000	0.624090000	-0.00 0.00 -0.00	-0.01 0.00 -0.02
H	-2.570692000	3.360436000	1.577773000	0.01 -0.01 0.00	0.15 -0.09 -0.00
H	-2.846890000	3.633693000	-0.172194000	-0.01 0.00 0.00	-0.14 0.05 0.04
H	-3.788148000	2.426123000	0.694251000	-0.00 0.00 -0.00	-0.02 0.04 -0.06
C	2.567125000	2.785900000	-1.236306000	-0.00 -0.00 -0.00	-0.01 -0.00 -0.02
H	3.402530000	2.774373000	-0.539407000	-0.00 -0.01 0.00	-0.06 -0.15 0.05
H	2.911785000	2.659626000	-2.260580000	0.00 0.01 -0.00	0.07 0.15 -0.00
H	2.076103000	3.758432000	-1.162504000	-0.00 -0.00 -0.00	-0.02 -0.00 -0.07
C	2.452000000	-2.905114000	-1.218491000	-0.00 0.00 -0.00	-0.01 -0.00 -0.02
H	3.214201000	-2.467070000	-1.865497000	-0.00 -0.00 -0.00	-0.02 -0.06 -0.06
H	2.930712000	-3.314323000	-0.332188000	-0.00 0.00 0.00	-0.12 0.10 0.10
H	1.955710000	-3.690862000	-1.786400000	0.01 -0.00 -0.00	0.12 -0.04 -0.06

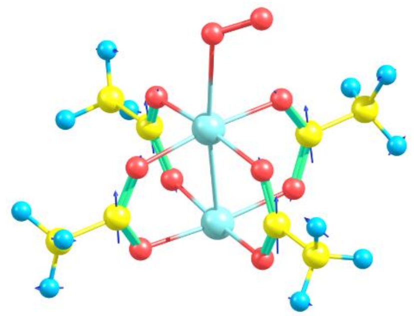

Table S9. Ideal-CO (singlet biradical)

32				CO vibration (2277 cm^{-1})	Assymetric OCO (1713 cm^{-1})	
C	-0.004897000	-4.021845000	-0.059437000	-0.00 0.00 0.00	0.00 -0.00 -0.02	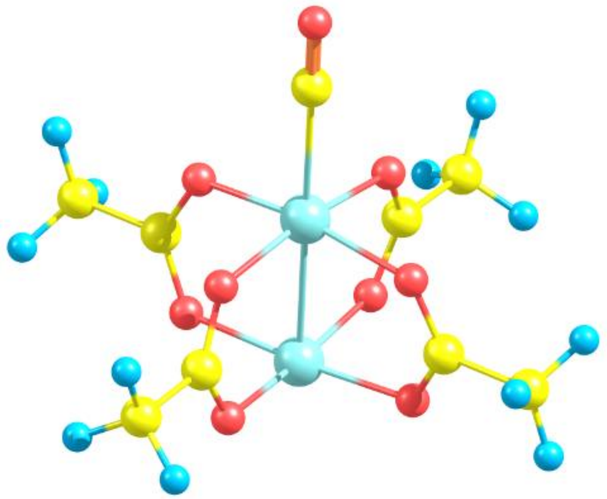
C	0.005817000	-2.522427000	-0.006470000	0.00 -0.00 -0.00	-0.03 -0.01 0.38	
O	-0.098289000	-1.980051000	1.121153000	-0.00 0.00 0.00	0.01 -0.06 -0.14	
Cu	-0.002958000	-0.015728000	1.340773000	0.00 0.00 -0.00	0.00 -0.00 0.00	
O	-1.966317000	0.084563000	1.141503000	0.00 0.00 0.00	-0.06 -0.01 -0.13	
C	-2.524020000	-0.004585000	0.018671000	-0.00 -0.00 -0.00	-0.01 0.03 0.38	
C	-4.024377000	-0.018863000	0.018819000	0.00 0.00 0.00	0.00 -0.00 -0.02	
O	0.113109000	-1.927794000	-1.108351000	-0.00 -0.00 0.00	0.01 0.07 -0.13	
Cu	0.006186000	0.021125000	-1.164695000	-0.00 0.00 -0.00	0.00 -0.00 0.00	
O	1.954253000	0.128263000	-1.067002000	0.00 -0.00 0.00	-0.06 0.01 -0.13	
C	2.527706000	0.006851000	0.044499000	0.00 0.00 -0.00	-0.00 -0.04 0.38	
C	4.027848000	-0.005504000	0.020208000	-0.00 -0.00 0.00	0.00 0.00 -0.02	
O	-1.944595000	-0.088432000	-1.091829000	-0.00 0.00 0.00	0.07 -0.01 -0.14	
O	-0.103896000	1.966910000	-1.051893000	0.00 0.00 0.00	-0.01 -0.06 -0.13	
C	-0.002123000	2.528911000	0.067510000	-0.00 0.00 -0.00	*0.03 -0.00 0.38*	
C	0.007215000	4.029240000	0.058571000	0.00 -0.00 0.00	-0.00 0.00 -0.02	
O	1.964000000	-0.110678000	1.160371000	-0.00 -0.00 0.00	0.06 0.01 -0.14	
O	0.096740000	1.953762000	1.179237000	0.00 -0.00 0.00	-0.01 0.07 -0.14	
H	4.389642000	0.862505000	-0.530876000	-0.00 0.00 -0.00	0.10 -0.06 -0.04	
H	4.438383000	-0.011593000	1.026799000	0.00 -0.00 0.00	-0.17 0.01 0.06	
H	4.364619000	-0.891983000	-0.519135000	-0.00 -0.00 0.00	0.07 0.05 -0.05	
H	-0.890286000	-4.349832000	-0.605836000	-0.00 0.00 0.00	0.05 -0.07 -0.05	
H	0.864137000	-4.371486000	-0.616848000	0.00 0.00 -0.00	-0.06 -0.10 -0.04	
H	-0.009914000	-4.451628000	0.939086000	-0.00 -0.00 0.00	0.01 0.17 0.07	
H	-4.369915000	-0.908380000	0.547344000	-0.00 0.00 0.00	0.08 -0.05 -0.05	
H	-4.394790000	0.846333000	0.568854000	-0.00 -0.00 0.00	0.10 0.06 -0.04	
H	-4.418463000	-0.017374000	-0.994313000	0.00 0.00 -0.00	-0.18 -0.01 0.06	
H	0.889158000	4.374423000	-0.482676000	0.00 -0.00 0.00	-0.05 0.08 -0.05	
H	-0.865328000	4.393667000	-0.483780000	-0.00 -0.00 -0.00	0.06 0.10 -0.04	
H	0.016437000	4.429496000	1.069256000	0.00 0.00 0.00	-0.01 -0.18 0.06	
C	-0.017474000	-0.051165000	3.661476000	-0.01 -0.01 0.80	-0.00 -0.00 0.01	
O	-0.027419000	-0.069357000	4.780320000	*0.01 0.01 -0.60*	0.00 0.00 -0.00	

Table S10. **I** - Defect (doublet)

23				One of three IR-active assymetic carboxylate vibrations (1626 cm^{-1})	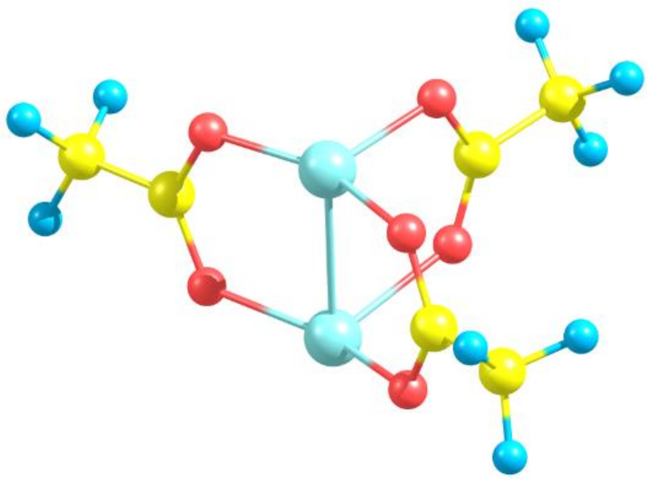
Cu	-0.000921000	-0.692667000	-1.225902000	0.00 0.00 -0.00	
Cu	-0.000712000	-0.068561000	1.156601000	-0.00 0.00 0.00	
O	1.835232000	-0.428656000	1.211760000	0.02 -0.02 -0.08	
C	2.426112000	-0.832011000	0.157159000	*0.16 0.10 0.42*	
O	1.872842000	-1.076758000	-0.930600000	-0.11 -0.05 -0.21	
O	-1.875514000	-1.072830000	-0.930773000	0.11 -0.05 -0.21	
O	0.000921000	1.465582000	-1.290937000	0.00 0.09 0.12	
C	-2.428745000	-0.826752000	0.156696000	*-0.16 0.10 0.42*	
O	-1.837395000	-0.424792000	1.211587000	-0.02 -0.02 -0.08	
O	0.001444000	1.843628000	0.911328000	0.00 0.00 0.03	
C	0.002268000	2.217699000	-0.310765000	*-0.00 -0.17 -0.21*	
C	-3.909352000	-0.999104000	0.272051000	0.04 0.00 -0.02	
H	-4.306714000	-1.527287000	-0.591078000	-0.26 0.03 0.11	
H	-4.153873000	-1.528846000	1.192351000	0.15 -0.12 -0.05	
H	-4.372446000	-0.012686000	0.335520000	0.06 0.02 -0.15	
C	0.009204000	3.707938000	-0.502798000	-0.00 0.03 0.00	
H	-0.840029000	4.150356000	0.018723000	0.05 0.06 0.06	
H	0.912631000	4.126766000	-0.057641000	-0.04 0.06 0.07	
H	-0.030513000	3.956236000	-1.560893000	-0.00 -0.15 -0.05	
C	3.906263000	-1.007870000	0.273191000	-0.04 0.00 -0.02	
H	4.302724000	-1.537107000	-0.589711000	0.26 0.03 0.11	
H	4.371692000	-0.022551000	0.336672000	-0.06 0.02 -0.15	
H	4.149151000	-1.538067000	1.193655000	-0.15 -0.12 -0.05	

Table S11. Defect-O_2 (doublet, coordination on Cu^{2+}, strongest O_2 adsorption on defect, see Table above, spin-densities on copper shown)

25				One of three IR-active assymetic carboxylate vibrations (1619 cm^{-1})	O-O stretching (1691 cm^{-1})	
Cu	0.006978000	-0.021450000	-1.078368000	-0.00 0.00 0.00	-0.00 0.00 -0.00	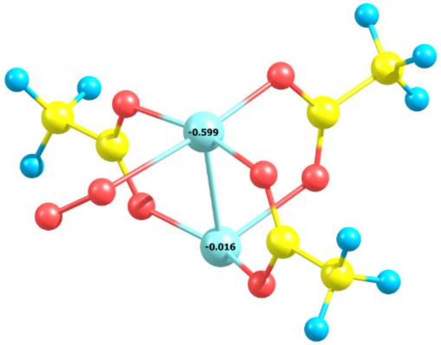
Cu	0.043201000	-0.175677000	1.379018000	0.00 -0.01 0.00	0.00 -0.00 0.00	
O	1.851350000	0.409546000	1.203850000	0.14 0.03 -0.25	-0.02 -0.00 0.04	
C	2.341393000	0.743936000	0.105578000	*-0.17 -0.03 0.51*	0.02 0.00 -0.08	
O	1.787410000	0.588913000	-1.026786000	-0.03 -0.01 -0.12	0.01 0.00 0.02	
O	-1.533099000	-1.106547000	-1.083583000	0.02 0.02 -0.10	-0.01 -0.01 0.03	
O	-0.886865000	1.679956000	-1.221356000	-0.01 -0.00 0.05	-0.01 0.01 0.03	
C	-1.973974000	-1.524102000	0.030885000	*0.13 -0.01 0.41*	-0.02 0.01 -0.11	
O	-1.393229000	-1.410349000	1.130429000	-0.11 -0.00 -0.19	0.03 0.00 0.05	
O	-1.083121000	1.661474000	1.005844000	0.00 -0.11 0.28	0.00 -0.04 0.09	
C	-1.286414000	2.159756000	-0.105395000	*-0.02 0.21 -0.47*	0.01 0.04 -0.16	
O	1.312483000	-2.543373000	-0.735447000	-0.01 0.00 -0.00	*-0.56 0.21 -0.33*	
O	2.436111000	-2.697488000	-0.361154000	0.01 -0.00 0.00	*0.56 -0.21 0.33*	
C	-3.310834000	-2.188897000	0.071505000	-0.03 -0.01 -0.03	0.00 -0.00 0.01	
H	-3.948056000	-1.517368000	0.654875000	0.00 0.00 0.00	0.00 0.00 0.00	
H	-3.766872000	-2.359260000	-0.906853000	0.00 0.00 0.00	0.00 0.00 0.00	
H	-3.189968000	-3.120549000	0.632008000	0.00 0.00 0.00	0.00 0.00 0.00	
C	-2.068418000	3.434124000	-0.246144000	0.02 -0.05 0.02	0.00 -0.01 0.01	
H	-2.322512000	3.853960000	0.730699000	0.00 0.00 0.00	0.00 0.00 0.00	
H	-1.474525000	4.134650000	-0.841412000	0.00 0.00 0.00	0.00 0.00 0.00	
H	-2.982592000	3.210561000	-0.804705000	0.00 0.00 0.00	0.00 0.00 0.00	
C	3.684237000	1.397002000	0.143786000	0.04 0.00 -0.04	-0.00 -0.00 0.01	
H	3.523557000	2.476825000	0.250229000	0.00 0.00 0.00	0.00 0.00 0.00	
H	4.194501000	1.029719000	1.035070000	0.00 0.00 0.00	0.00 0.00 0.00	
H	4.267021000	1.220163000	-0.764442000	0.00 0.00 0.00	0.00 0.00 0.00	

Table S12. **II** - Defect-CO on Cu^+ (doublet, strongest CO adsorption on defect)

25				CO vibration (2226 cm^{-1})		
Cu	-0.124175000	-1.229568000	-0.888487000	-0.00	-0.00	-0.00
Cu	0.103146000	1.312842000	0.254363000	-0.00	-0.00	0.00
O	-1.934159000	0.890812000	-0.191901000	0.00	0.00	0.00
C	-2.541214000	-0.059818000	-0.700930000	-0.00	-0.01	-0.00
O	-1.964461000	-1.066267000	-1.240861000	0.00	0.00	0.00
O	1.740942000	-1.457626000	-1.042442000	0.00	0.00	0.00
O	-0.313995000	-1.952369000	0.869297000	0.00	0.00	0.00
C	2.434413000	-0.398985000	-0.855703000	0.00	-0.01	-0.00
O	1.939664000	0.726019000	-0.705302000	-0.00	0.00	0.00
O	0.316899000	-0.008121000	1.780766000	0.00	0.01	-0.00
C	0.005574000	-1.203898000	1.845671000	-0.00	-0.01	0.00
O	0.319496000	4.303916000	0.345444000	*-0.04*	*-0.59*	*-0.00*
C	0.239938000	3.181456000	0.345238000	*0.06*	*0.81*	*0.00*
C	3.940997000	-0.548455000	-0.808073000	-0.00	0.00	-0.00
H	4.273214000	-0.542213000	0.245654000	0.00	0.00	0.00
H	4.301353000	-1.480023000	-1.270724000	0.00	0.00	0.00
H	4.388832000	0.328946000	-1.294753000	0.00	0.00	0.00
C	-0.002769000	-1.884310000	3.178742000	0.00	0.00	0.00
H	0.166122000	-1.137646000	3.953354000	0.00	0.00	0.00
H	-0.958064000	-2.397404000	3.315238000	0.00	0.00	0.00
H	0.788133000	-2.639871000	3.188910000	0.00	0.00	0.00
C	-4.055883000	-0.058329000	-0.701426000	0.00	0.00	0.00
H	-4.449921000	-0.787858000	0.029280000	0.00	0.00	0.00
H	-4.421759000	0.942524000	-0.431279000	0.00	0.00	0.00
H	-4.439482000	-0.358259000	-1.687209000	0.00	0.00	0.00

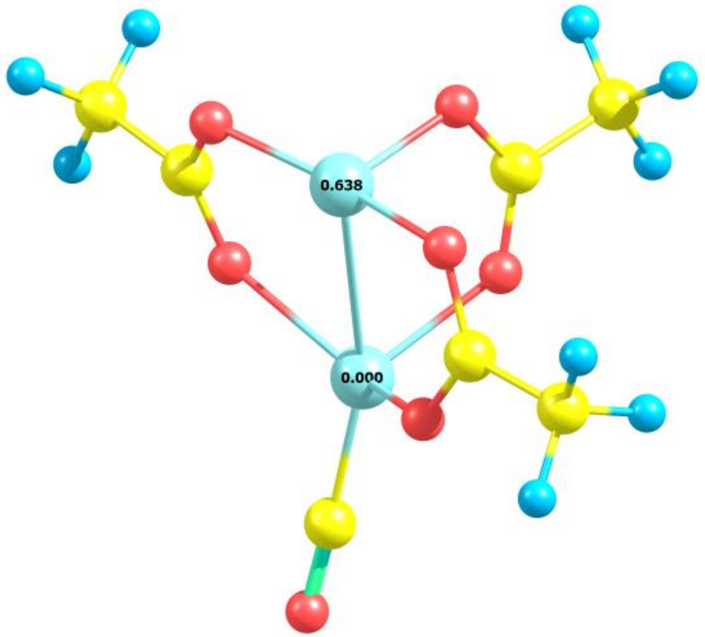

Table S13. Defect-CO on Cu^{2+}

25				CO vibration (2282 cm^{-1})			
Cu	0.072379000	-0.539360000	-0.890958000	0.00	-0.00	0.00	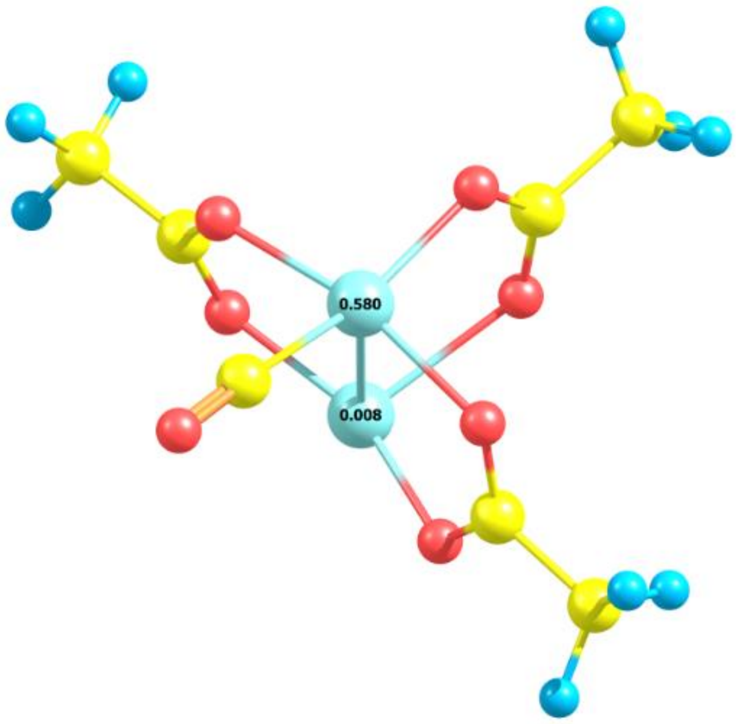
Cu	0.020367000	0.354348000	1.481884000	0.00	0.00	0.00	
O	1.916559000	0.268556000	1.393814000	0.00	0.00	0.00	
C	2.522135000	-0.161090000	0.392980000	-0.00	-0.00	-0.00	
O	1.996793000	-0.618837000	-0.664900000	-0.00	0.00	0.00	
O	-1.859708000	-0.677425000	-0.710816000	0.00	-0.00	0.00	
O	0.045994000	1.239458000	-1.584123000	-0.00	0.00	-0.00	
C	-2.362894000	-0.571245000	0.446484000	0.00	-0.00	-0.00	
O	-1.733733000	-0.395774000	1.509435000	-0.00	-0.00	0.00	
O	-0.466737000	2.190688000	0.372459000	-0.00	0.00	0.00	
C	-0.285220000	2.230936000	-0.844614000	0.00	-0.00	-0.00	
C	0.118070000	-2.640618000	-1.025028000	*-0.01*	*0.80*	*-0.04*	
O	0.138377000	-3.756399000	-0.962479000	*0.01*	*-0.59*	*0.03*	
C	-3.847346000	-0.628903000	0.561024000	-0.00	-0.00	0.00	
H	-4.161303000	0.401834000	0.743103000	0.00	0.00	0.00	
H	-4.321656000	-1.001808000	-0.345343000	0.00	0.00	0.00	
H	-4.099038000	-1.228843000	1.437715000	0.00	0.00	0.00	
C	-0.471568000	3.521140000	-1.600555000	-0.00	0.00	-0.00	
H	-0.544428000	4.363839000	-0.905893000	0.00	0.00	0.00	
H	0.351799000	3.659825000	-2.309698000	0.00	0.00	0.00	
H	-1.398913000	3.440917000	-2.180729000	0.00	0.00	0.00	
C	4.011131000	-0.102131000	0.441510000	0.00	0.00	0.00	
H	4.282308000	0.905073000	0.115798000	0.00	0.00	0.00	
H	4.323132000	-0.236948000	1.477593000	0.00	0.00	0.00	
H	4.472257000	-0.829204000	-0.227332000	0.00	0.00	0.00	

Table S14. Defect with 2 CO on Cu^{+}

27				First of 2 CO vibrations (2223 cm^{-1})	Second CO-related vibration (2253 cm^{-1})	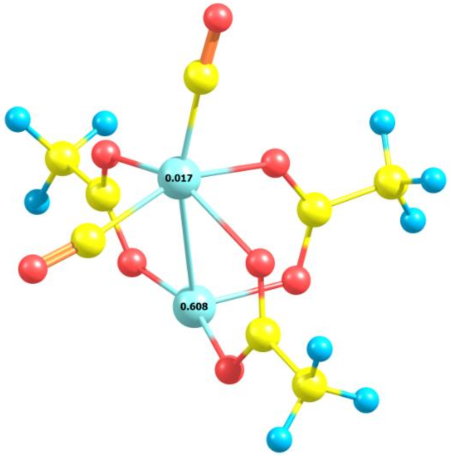
Cu	0.108800000	1.241735000	0.258010000	-0.00 0.00 -0.00	-0.00 -0.00 -0.00	
Cu	-0.121541000	-1.216683000	-0.904391000	0.00 -0.00 0.00	-0.00 -0.00 -0.00	
O	1.730801000	-1.395767000	-1.117912000	-0.00 -0.00 -0.00	-0.00 0.00 0.00	
C	2.518557000	-0.468215000	-0.716146000	0.00 0.00 0.00	0.00 -0.01 -0.00	
O	2.163657000	0.635092000	-0.294847000	-0.00 -0.00 -0.00	-0.00 0.00 0.00	
O	-2.040657000	0.649485000	0.257663000	0.00 -0.00 -0.00	0.00 0.00 0.00	
O	0.300376000	-0.331035000	1.733457000	-0.00 -0.01 0.00	0.00 0.00 -0.00	
C	-2.577720000	-0.160722000	-0.501029000	0.00 0.00 0.00	-0.00 -0.00 -0.00	
O	-1.945222000	-0.976159000	-1.263816000	0.00 -0.00 -0.00	0.00 0.00 0.00	
O	-0.332468000	-2.219592000	0.727744000	-0.00 -0.00 -0.00	0.00 0.00 -0.00	
C	-0.031683000	-1.522692000	1.746058000	0.00 0.01 0.00	-0.00 -0.00 0.00	
C	-0.074347000	1.803220000	-1.646748000	*0.01 -0.27 0.68*	*-0.00 0.12 -0.31*	
O	-0.085810000	2.213759000	-2.692150000	*-0.01 0.20 -0.51*	*0.00 -0.09 0.23*	
C	-4.080384000	-0.215146000	-0.537454000	-0.00 -0.00 -0.00	0.00 0.00 0.00	
H	-4.427303000	-1.090426000	0.030812000	-0.00 -0.00 -0.00	0.00 -0.00 0.00	
H	-4.441055000	0.701547000	-0.062893000	0.00 -0.00 0.00	-0.00 0.00 0.00	
H	-4.468747000	-0.312587000	-1.556797000	-0.00 -0.00 0.00	0.00 0.00 -0.00	
C	-0.105851000	-2.224019000	3.071070000	-0.00 -0.00 0.00	0.00 0.00 -0.00	
H	-0.126511000	-3.311122000	2.964211000	-0.00 0.00 0.00	0.00 -0.00 -0.00	
H	0.750455000	-1.892113000	3.659663000	0.00 0.00 -0.00	-0.00 0.00 0.00	
H	-1.017320000	-1.882503000	3.572382000	-0.00 0.00 -0.00	0.00 0.00 -0.00	
C	3.996706000	-0.753821000	-0.757340000	-0.00 -0.00 -0.00	-0.00 0.00 0.00	
H	4.310347000	-1.157568000	0.217690000	0.00 0.00 0.00	-0.00 0.00 0.00	
H	4.285155000	-1.473231000	-1.531439000	0.00 0.00 0.00	-0.00 -0.00 -0.00	
H	4.483819000	0.213433000	-0.905195000	-0.00 -0.00 0.00	0.00 0.00 0.00	
C	0.283678000	2.597007000	1.626947000	*0.03 0.22 0.25*	*0.07 0.47 0.56*	
O	0.390184000	3.317267000	2.480416000	*-0.02 -0.16 -0.19*	*-0.05 -0.34 -0.41*	

Table S15. Defect with 2 CO molecules on different Cu atoms, doublet

27				CO on Cu^{+} (2220 cm-1)	CO on Cu^{2+} (2283 cm^{-1})
Cu	-0.469225000	-1.355039000	-0.096274000	-0.00 -0.00 -0.00	-0.00 -0.00 -0.00
Cu	0.432485000	1.397838000	0.021568000	-0.00 -0.00 0.00	0.00 0.00 -0.00
O	-1.606684000	1.190114000	-0.424990000	0.01 0.00 0.00	0.00 0.00 -0.00
C	-2.521024000	0.372589000	-0.220607000	-0.01 -0.01 0.00	-0.00 -0.01 0.00
O	-2.350564000	-0.876282000	-0.075396000	0.00 0.00 -0.00	0.00 0.00 -0.00
O	1.379611000	-1.941988000	-0.245547000	0.00 0.00 -0.00	-0.00 0.00 -0.00
O	-0.381492000	-1.372478000	1.819395000	0.00 0.00 0.00	0.00 0.00 0.00
C	2.094593000	-1.033607000	-0.766639000	0.00 -0.01 0.00	-0.00 -0.00 0.00
O	1.649412000	0.067558000	-1.145741000	-0.00 0.00 0.00	0.00 0.00 0.00
O	0.671509000	0.598726000	1.863967000	0.00 0.01 -0.01	0.00 0.00 0.00
C	0.230005000	-0.430980000	2.402997000	-0.01 -0.01 0.00	-0.00 -0.00 -0.00
O	1.300924000	4.168075000	-0.689360000	*-0.17 -0.54 0.15*	0.00 0.02 -0.00
C	0.974576000	3.128018000	-0.406312000	*0.23 0.75 -0.20*	-0.01 -0.02 0.01
C	-0.635336000	-1.399030000	-2.189539000	0.00 -0.00 0.02	*0.07 -0.08 0.80*
O	-0.736392000	-1.280574000	-3.296548000	-0.00 0.00 -0.02	*-0.05 0.06 -0.59*
C	3.562270000	-1.314858000	-0.911406000	-0.00 0.00 -0.00	0.00 -0.00 -0.00
H	4.068939000	-0.824339000	-0.070343000	-0.00 0.00 -0.00	0.00 -0.00 -0.00
H	3.774212000	-2.388149000	-0.875443000	-0.00 -0.00 0.00	0.00 0.00 0.00
H	3.929844000	-0.861846000	-1.838023000	0.00 0.00 0.00	0.00 -0.00 0.00
C	0.434329000	-0.619113000	3.880351000	0.00 0.00 -0.00	0.00 0.00 0.00
H	0.779604000	0.312514000	4.336471000	0.00 -0.00 0.00	-0.00 -0.00 0.00
H	-0.498282000	-0.966816000	4.337091000	-0.00 0.00 -0.00	-0.00 0.00 -0.00
H	1.187354000	-1.403041000	4.023950000	0.00 0.00 -0.00	0.00 0.00 0.00
C	-3.934251000	0.873514000	-0.120005000	0.00 0.00 -0.00	-0.00 0.00 -0.00
H	-4.135140000	1.083075000	0.938626000	0.00 0.00 -0.00	-0.00 0.00 -0.00
H	-4.035052000	1.806627000	-0.681865000	-0.00 0.00 0.00	-0.00 -0.00 0.00
H	-4.647599000	0.116399000	-0.463256000	0.00 -0.00 -0.00	0.00 0.00 0.00

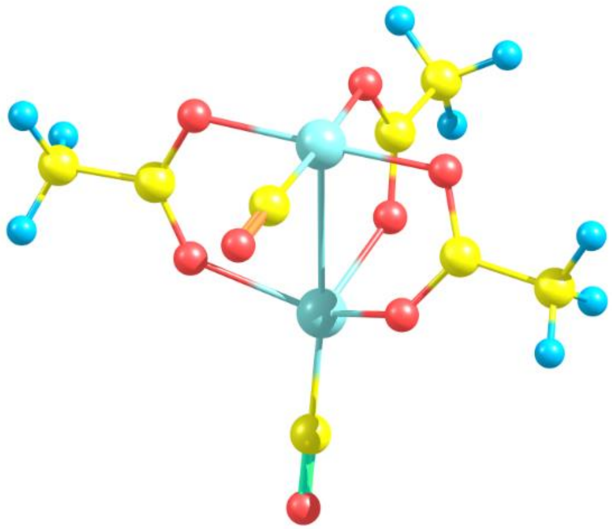

Table S16. **III** – coabsorption of O_2 (on Cu^{2+}) and CO (on Cu^{+}), quartet

27				O_2-stretching (1706 cm^{-1})	OCO-assymetric (1709 cm^{-1})	CO-vibration (534 cm^{-1})
Cu	-0.349735000	-0.368757000	1.316979000	-0.00 0.00 0.00	-0.00 -0.00 0.01	0.00 0.00 -0.00
Cu	0.548320000	0.493047000	-1.238220000	-0.00 -0.00 0.00	0.00 -0.00 0.00	0.00 0.00 -0.00
O	-1.187019000	1.504796000	-0.681206000	0.07 -0.01 -0.09	0.14 -0.02 -0.20	-0.00 0.00 0.00
C	-1.886164000	1.585376000	0.335433000	-0.10 -0.01 0.18	*-0.21 -0.03 0.40*	0.01 0.00 -0.01
O	-1.726663000	0.881247000	1.389239000	0.00 0.02 -0.04	-0.00 0.04 -0.09	-0.00 -0.00 0.00
O	0.470965000	-2.037551000	1.146004000	0.00 -0.00 -0.03	0.01 -0.01 -0.09	-0.00 -0.00 0.00
O	1.062146000	0.614031000	2.153690000	0.03 0.02 -0.03	0.07 0.03 -0.07	-0.00 -0.00 0.00
C	0.669678000	-2.355605000	-0.075257000	0.02 -0.06 0.13	*0.04 -0.17 0.34*	-0.00 0.01 -0.01
O	0.432806000	-1.610444000	-1.035849000	-0.01 0.05 -0.06	-0.03 0.12 -0.16	0.00 -0.01 0.00
O	1.966375000	1.003405000	0.148229000	-0.00 -0.00 -0.10	-0.00 -0.01 -0.22	0.00 0.00 0.01
C	1.969357000	1.062256000	1.385776000	-0.04 -0.01 0.19	*-0.08 -0.03 0.41*	0.00 -0.00 -0.01
O	1.136941000	1.061224000	-4.123468000	0.00 0.00 -0.00	0.00 0.00 -0.01	*0.11 0.11 -0.57*
C	0.925831000	0.854629000	-3.037183000	-0.00 -0.00 0.01	-0.00 -0.00 0.01	*-0.15 -0.15 0.78*
C	1.228110000	-3.725646000	-0.302565000	-0.00 0.01 -0.01	-0.01 0.02 -0.02	0.00 -0.00 0.00
H	2.269098000	-3.737311000	0.026121000	0.00 0.03 -0.02	0.01 0.07 -0.06	0.00 -0.00 0.00
H	0.692064000	-4.457339000	0.301397000	-0.04 0.03 -0.01	-0.10 0.08 -0.02	0.00 -0.00 -0.00
H	1.182570000	-3.991106000	-1.355957000	0.03 -0.06 0.02	0.09 -0.16 0.04	-0.00 0.00 0.00
C	3.135250000	1.695158000	2.089927000	-0.00 -0.00 -0.01	-0.01 -0.01 -0.03	0.00 0.00 0.00
H	3.755584000	2.244101000	1.385308000	0.06 0.02 0.07	0.12 0.04 0.15	-0.00 -0.00 -0.00
H	2.784947000	2.351811000	2.885451000	-0.05 0.01 -0.04	-0.11 0.02 -0.09	0.00 0.00 0.00
H	3.730708000	0.908750000	2.556978000	0.00 -0.02 -0.04	0.01 -0.04 -0.09	0.00 0.00 0.00
C	-3.035095000	2.545249000	0.379197000	0.01 -0.00 -0.01	0.03 -0.01 -0.02	-0.00 0.00 0.00
H	-2.997803000	3.130041000	1.298079000	0.07 -0.00 -0.01	0.15 -0.01 -0.02	-0.00 0.00 -0.00
H	-3.026970000	3.198684000	-0.489620000	-0.09 0.05 0.04	-0.19 0.09 0.08	0.00 -0.00 0.00
H	-3.967890000	1.978060000	0.397247000	0.02 -0.03 -0.04	0.04 -0.05 -0.09	-0.00 0.00 0.00
O	-2.568526000	-1.901320000	0.102205000	*-0.30 0.26 -0.51*	0.09 -0.08 0.16	-0.00 0.00 -0.00
O	-3.114911000	-1.415212000	-0.841464000	*0.29 -0.26 0.51*	-0.09 0.08 -0.16	0.00 -0.00 0.00

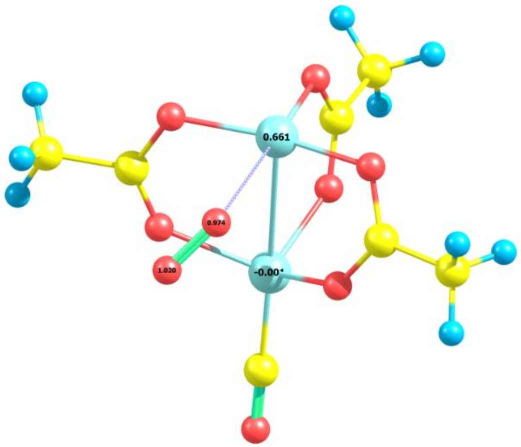

Table S17. Defect with 2 CO molecules absorbed on different Cu and physisorbed O_2 (quartet)

29				CO vibration on Cu^{+} (2222 cm-1)	CO vibration on Cu^{2+} (2287 cm-1)
Cu	0.613203000	-0.574275000	-1.183722000	0.00 -0.00 -0.00	-0.00 0.00 -0.00
Cu	-0.287060000	0.402757000	1.363325000	0.00 -0.00 -0.00	-0.00 0.00 0.00
O	-1.311300000	-1.346496000	0.913100000	0.00 0.01 0.00	-0.00 0.00 0.00
C	-1.314803000	-2.166640000	-0.019528000	0.00 -0.01 -0.01	0.00 -0.00 -0.01
O	-0.687004000	-2.021362000	-1.114274000	-0.00 0.00 0.00	0.00 0.00 0.00
O	1.858819000	0.924348000	-1.391373000	-0.00 0.00 0.00	-0.00 0.00 0.00
O	1.869690000	-1.563211000	-0.133514000	-0.00 0.00 0.00	0.00 -0.00 0.00
C	1.413261000	1.957524000	-0.794165000	0.01 -0.00 -0.00	0.00 -0.00 -0.00
O	0.340362000	1.972464000	-0.166885000	-0.00 -0.00 0.00	-0.00 0.00 0.00
O	1.561920000	-0.310541000	1.691449000	-0.01 0.00 0.00	-0.00 0.00 0.00
C	2.161827000	-1.179078000	1.037009000	0.01 -0.01 -0.01	-0.00 -0.00 -0.00
O	-1.829255000	2.349261000	3.030810000	*0.31 -0.39 -0.31*	-0.01 0.01 0.01
C	-1.240583000	1.598316000	2.433317000	*-0.42 0.54 0.43*	0.01 -0.01 -0.01
C	2.267249000	3.201187000	-0.897649000	-0.00 -0.00 -0.00	-0.00 0.00 0.00
H	3.032832000	3.193354000	-0.104543000	0.00 0.00 0.00	0.00 0.00 0.00
H	2.780254000	3.217315000	-1.869556000	0.00 0.00 0.00	0.00 0.00 0.00
H	1.657024000	4.102028000	-0.757698000	0.00 0.00 0.00	0.00 0.00 0.00
C	3.351634000	-1.853331000	1.652953000	-0.00 0.00 0.00	0.00 -0.00 0.00
H	3.409089000	-1.592100000	2.710944000	0.00 0.00 0.00	0.00 0.00 0.00
H	3.268473000	-2.935099000	1.513229000	0.00 0.00 0.00	0.00 0.00 0.00
H	4.252767000	-1.518956000	1.126390000	0.00 0.00 0.00	0.00 0.00 0.00
C	-2.119005000	-3.440086000	0.119747000	-0.00 0.00 0.00	-0.00 0.00 0.00
H	-1.437884000	-4.295470000	0.256056000	0.00 0.00 0.00	0.00 0.00 0.00
H	-2.790782000	-3.376358000	0.985421000	0.00 0.00 0.00	0.00 0.00 0.00
H	-2.686692000	-3.624286000	-0.802701000	0.00 0.00 0.00	0.00 0.00 0.00
O	-1.086591000	0.838114000	-3.503253000	-0.01 0.01 -0.01	*-0.31 0.29 -0.42*
C	-0.507753000	0.297552000	-2.714864000	0.01 -0.01 0.01	*0.42 -0.39 0.57*
O	-3.079546000	0.546010000	-0.949009000	0.00 -0.00 -0.00	-0.00 0.00 -0.00
O	-3.263868000	1.275279000	-0.022919000	-0.00 0.00 0.00	-0.00 0.00 0.00

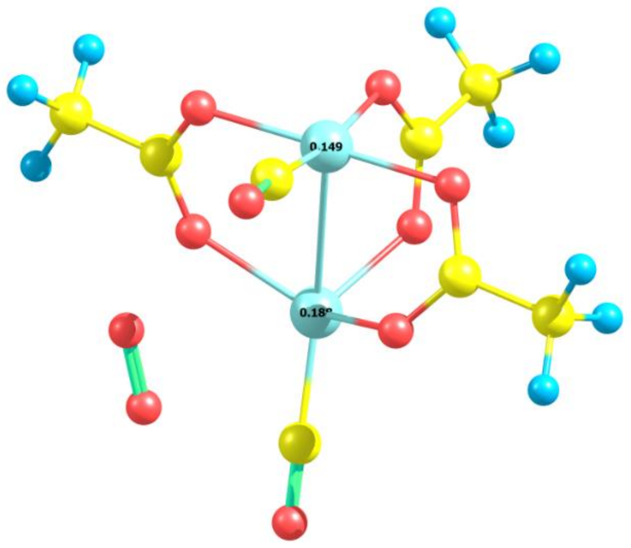

Table S18. **TS-I** - Transition state of $CO+O_2$ process – M06 geometry (doublet)

27				Imaginary frequency (321 cm^{-1})		
Cu	-0.034222000	-0.330906000	-1.160063000	0.00	0.01	-0.06
Cu	0.100293000	0.249483000	1.272504000	0.01	0.04	-0.04
O	2.030400000	-0.084388000	1.109784000	-0.03	-0.03	0.01
C	2.485641000	-0.655836000	0.089482000	0.03	-0.02	-0.02
O	1.821663000	-0.996716000	-0.924899000	0.09	-0.00	-0.00
O	-1.964314000	0.163835000	-0.974701000	-0.07	0.08	-0.00
O	0.547388000	1.572917000	-1.388286000	0.02	0.03	-0.00
C	-2.459629000	0.420884000	0.154281000	-0.02	0.02	-0.02
O	-1.837590000	0.419640000	1.246378000	0.02	-0.02	0.00
O	0.324785000	2.115543000	0.769719000	0.00	0.01	0.00
C	0.543017000	2.390317000	-0.441416000	0.01	0.01	-0.01
O	0.004515000	-1.699096000	1.648007000	-0.03	-0.04	0.21
O	-0.849642000	-2.361967000	1.007832000	-0.01	-0.09	0.61
C	-0.720260000	-2.229385000	-0.904639000	-0.01	-0.03	-0.65
O	-1.179874000	-3.058746000	-1.524869000	-0.10	-0.22	-0.23
C	-3.927320000	0.774157000	0.220488000	-0.01	0.00	0.01
H	-4.015101000	1.806721000	0.585993000	0.00	0.00	0.00
H	-4.415239000	0.687693000	-0.758573000	0.00	0.00	0.00
H	-4.423641000	0.126666000	0.957950000	0.00	0.00	0.00
C	0.802657000	3.833590000	-0.768561000	0.00	0.01	0.00
H	0.997842000	4.397106000	0.146682000	0.00	0.00	0.00
H	1.641334000	3.904875000	-1.468872000	0.00	0.00	0.00
H	-0.085484000	4.238313000	-1.269533000	0.00	0.00	0.00
C	3.972114000	-0.937695000	0.077919000	0.02	0.00	0.01
H	4.492375000	-0.019951000	-0.233490000	0.00	0.00	0.00
H	4.307411000	-1.189000000	1.092954000	0.00	0.00	0.00
H	4.228481000	-1.735517000	-0.630936000	0.00	0.00	0.00

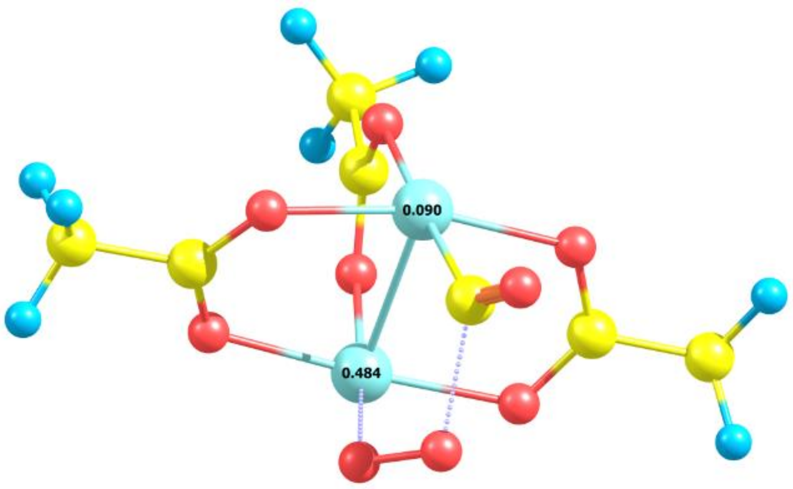

Table S19. **TS-I** - Transition state of $CO+O_2$ process – CASSCF geometry – (3 electrons on 3 orbitals, averaging over doublet and quartet multiplicity)

27				Imaginary frequency (289 cm^{-1})		
Cu	-0.070520000	-0.233691000	-1.322403000	0.010750	0.039309	0.065191
Cu	0.134777000	0.496416000	1.436536000	-0.014082	-0.065960	0.006441
O	1.967490000	-0.075605000	1.489742000	0.045243	0.057398	-0.042869
C	2.448304000	-0.526757000	0.426362000	-0.010811	-0.004832	-0.018907
O	1.815952000	-0.591248000	-0.636154000	-0.029023	-0.039676	-0.030363
O	-1.906400000	0.257666000	-0.581524000	0.008155	-0.054033	-0.031431
O	0.343131000	1.613617000	-1.757883000	-0.000582	0.003020	-0.002531
C	-2.420490000	0.577813000	0.498476000	0.006699	-0.013501	-0.020125
O	-1.763502000	0.770651000	1.545588000	-0.017713	0.065653	-0.042459
O	0.493377000	2.150505000	0.362254000	-0.002130	-0.007426	-0.002120
C	0.539723000	2.436700000	-0.844264000	-0.000166	0.003367	0.010534
O	-0.356313000	-1.642818000	1.111850000	*0.080863*	*0.247176*	*0.186998*
O	-0.594970000	-2.640155000	0.389640000	*-0.001918*	*0.084036*	*0.508461*
C	-0.542245000	-2.297009000	-1.377075000	*-0.019536*	*-0.034600*	*-0.706652*
O	-0.769355000	-3.258812000	-1.923190000	*-0.038471*	*-0.174061*	*-0.226168*
C	-3.913478000	0.713332000	0.577003000	0.003360	-0.022737	-0.032698
H	-4.190553000	1.437624000	1.338559000	0.005377	-0.033578	-0.022030
H	-4.322098000	0.985277000	-0.392618000	0.002646	-0.003856	-0.027920
H	-4.318340000	-0.259232000	0.863555000	0.005336	-0.027796	-0.047877
C	0.814719000	3.863352000	-1.226837000	-0.002091	0.003975	0.009046
H	1.456333000	4.335867000	-0.487359000	-0.005884	0.003808	0.012384
H	1.254852000	3.914868000	-2.219055000	0.001377	0.010200	0.010732
H	-0.138761000	4.394923000	-1.242060000	-0.003290	0.001758	0.007754
C	3.857082000	-1.043963000	0.462927000	-0.015032	-0.022612	-0.030114
H	4.441071000	-0.504411000	1.204052000	-0.011550	-0.029658	-0.027906
H	3.815463000	-2.094622000	0.756628000	-0.031067	-0.022355	-0.031823
H	4.312352000	-0.977752000	-0.521614000	-0.010163	-0.024791	-0.028583

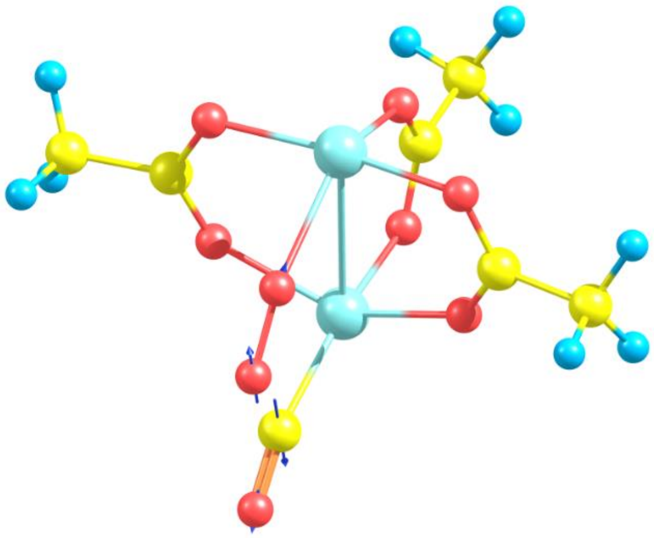

Table S20. **TS-I** - Transition state of CO+O_2 process – B3LYP geometry (doublet)

27				Imaginary frequency (285 cm^{-1})		
Cu	-0.085183000	-0.324981000	-1.165228000	0.00	0.01	-0.07
Cu	0.132940000	0.295415000	1.305619000	0.02	0.04	-0.04
O	2.019907000	-0.303221000	1.098286000	-0.03	-0.04	0.02
C	2.390414000	-0.912452000	0.062133000	0.02	-0.02	-0.02
O	1.682558000	-1.135556000	-0.967494000	0.08	0.01	-0.02
O	-1.942924000	0.298368000	-1.012233000	-0.06	0.08	-0.01
O	0.653615000	1.553449000	-1.369340000	0.02	0.04	0.00
C	-2.422390000	0.640769000	0.111754000	-0.01	0.02	-0.02
O	-1.791799000	0.680683000	1.201620000	0.01	-0.03	0.01
O	0.613411000	2.130463000	0.798970000	0.01	0.01	0.00
C	0.813632000	2.374370000	-0.428220000	0.01	0.01	-0.01
O	-0.210701000	-1.625439000	1.727153000	*-0.04*	*-0.04*	*0.22*
O	-1.122662000	-2.230465000	1.059257000	*-0.05*	*-0.06*	*0.64*
C	-0.909558000	-2.169703000	-0.841974000	*-0.01*	*-0.10*	*-0.61*
O	-1.393051000	-2.999987000	-1.451989000	*-0.10*	*-0.23*	*-0.25*
C	-3.888085000	1.010281000	0.119295000	-0.01	0.00	0.01
H	-4.116527000	1.664995000	0.964988000	0.00	0.00	0.00
H	-4.131732000	1.481685000	-0.836596000	0.00	0.00	0.00
H	-4.473207000	0.084196000	0.211175000	0.00	0.00	0.00
C	1.313717000	3.757270000	-0.791116000	0.00	0.01	0.00
H	1.042899000	4.491824000	-0.024870000	0.00	0.00	0.00
H	2.408331000	3.703839000	-0.863481000	0.00	0.00	0.00
H	0.915493000	4.033221000	-1.771628000	0.00	0.00	0.00
C	3.794795000	-1.475235000	0.023428000	0.01	-0.00	0.01
H	4.153251000	-1.466871000	-1.009089000	0.00	0.00	0.00
H	4.459856000	-0.915449000	0.688233000	0.00	0.00	0.00
H	3.734685000	-2.518194000	0.364279000	0.00	0.00	0.00

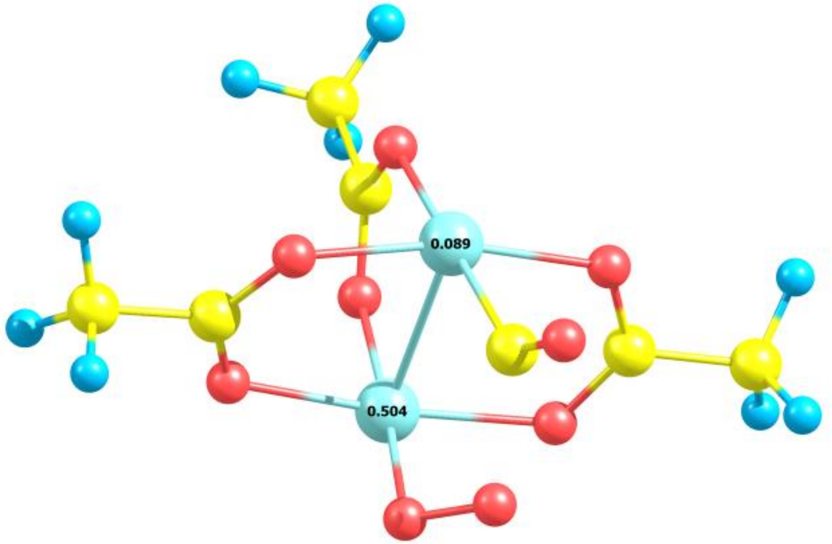

Table S21. Transition state of CO+O_2 process in presence of another CO molecule absorbed on same Cu where O_2 adsorbed. (doublet)

29				Imaginary frequency (251 cm^{-1})		
Cu	-0.531357000	0.676956000	-0.654025000	0.01	0.01	-0.07
Cu	0.738404000	-1.007501000	0.790506000	-0.02	0.03	-0.05
O	2.154333000	0.360261000	0.940321000	-0.03	-0.09	-0.08
C	2.087401000	1.408797000	0.243403000	-0.01	-0.01	-0.03
O	1.164573000	1.706881000	-0.549641000	0.00	0.03	0.02
O	-1.956217000	-0.752526000	-0.580088000	-0.03	0.00	0.03
O	-1.176139000	1.458751000	1.034078000	0.01	-0.01	-0.01
C	-1.729372000	-1.907406000	-0.158404000	0.00	0.03	-0.00
O	-0.642212000	-2.321666000	0.329086000	0.07	0.06	-0.02
O	-0.351703000	-0.211063000	2.272103000	0.04	-0.00	-0.03
C	-1.009541000	0.840007000	2.119034000	0.01	0.01	-0.02
C	-2.847684000	-2.922443000	-0.216099000	0.02	0.00	0.01
H	-3.198790000	-3.109611000	0.808286000	0.00	0.00	0.00
H	-3.681587000	-2.564475000	-0.831813000	0.00	0.00	0.00
H	-2.454253000	-3.869563000	-0.604505000	0.00	0.00	0.00
C	-1.695713000	1.427238000	3.320800000	0.01	-0.01	-0.01
H	-1.322163000	0.977327000	4.244547000	0.00	0.00	0.00
H	-1.557145000	2.512864000	3.320805000	0.00	0.00	0.00
H	-2.768339000	1.226193000	3.219307000	0.00	0.00	0.00
C	3.219185000	2.393761000	0.409637000	-0.01	-0.02	0.01
H	2.948297000	3.089738000	1.216744000	0.00	0.00	0.00
H	4.131652000	1.865301000	0.706090000	0.00	0.00	0.00
H	3.376024000	2.975146000	-0.507984000	0.00	0.00	0.00
C	-1.735628000	2.159825000	-1.917965000	-0.01	0.00	0.01
O	-2.348112000	2.925900000	-2.456500000	0.00	-0.00	-0.01
O	0.134661000	-0.391453000	-2.227325000	*0.04*	*-0.11*	*0.23*
O	1.334528000	-0.735101000	-2.261458000	*0.17*	*-0.26*	*0.54*
C	1.926056000	-1.784073000	-0.674435000	*-0.29*	*0.36*	*-0.40*
O	2.840504000	-2.441404000	-0.786235000	*-0.27*	*0.25*	*-0.04*

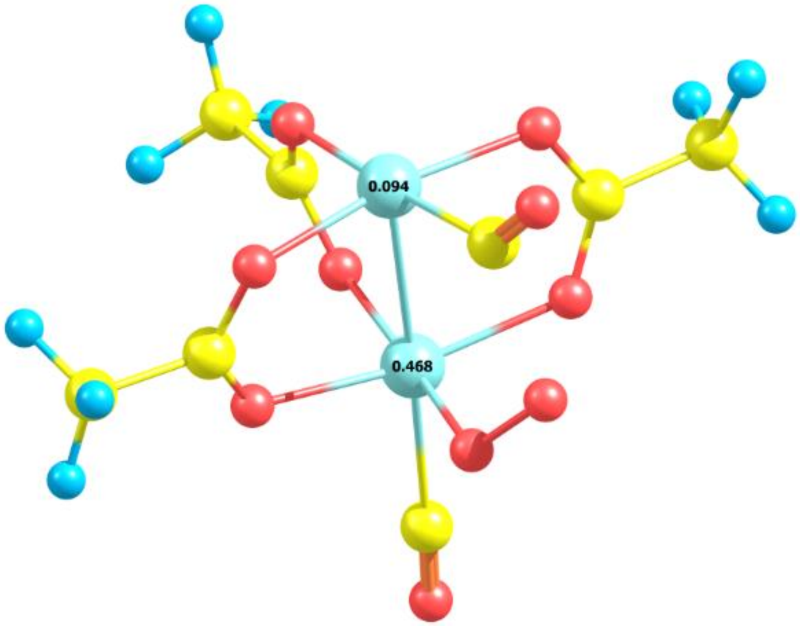

Table S22. Transition state of CO+O_2 process in presence of another CO molecule absorbed on same Cu where first CO adsorbed. (doublet)

29				Imaginary frequency (324 cm^{-1})		
Cu	-0.003845000	0.833743000	0.487707000	-0.01 0.03 -0.04		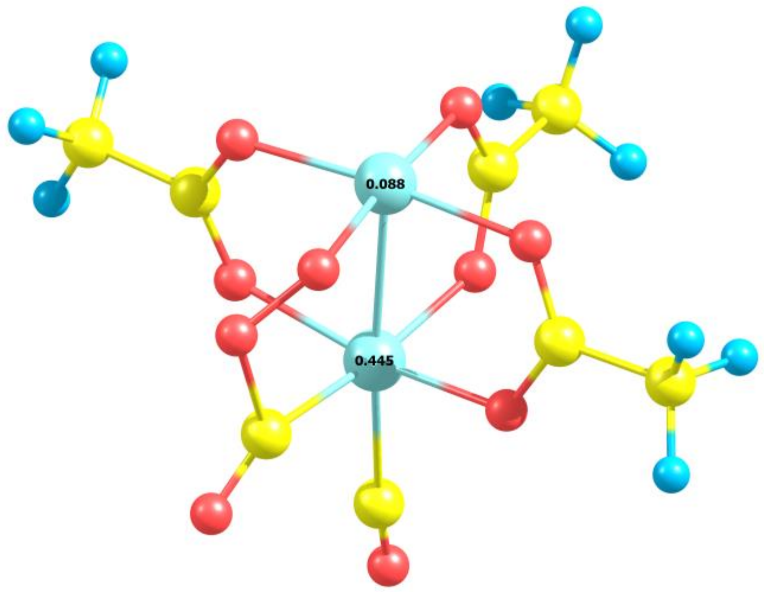
Cu	0.060787000	-1.223382000	-0.992267000	-0.00 0.03 0.02		
O	1.990335000	-0.958462000	-1.125297000	-0.01 -0.02 0.02		
C	2.502682000	0.110203000	-0.710555000	-0.02 0.00 0.00		
O	1.894970000	1.045377000	-0.131000000	-0.01 0.01 -0.02		
O	-1.950555000	0.297257000	0.651606000	0.00 0.01 -0.02		
O	0.529098000	-0.456158000	1.900150000	0.00 -0.00 0.01		
C	-2.469303000	-0.604399000	-0.053867000	0.01 0.01 0.01		
O	-1.864969000	-1.317235000	-0.895487000	0.03 -0.01 0.01		
O	0.261457000	-2.300415000	0.663280000	0.00 0.02 -0.03		
C	0.492936000	-1.704017000	1.739902000	0.00 0.02 0.00		
O	-0.035943000	-0.055954000	-2.551256000	*-0.04 0.09 0.15*		
O	-0.847970000	0.919303000	-2.477921000	*0.06 0.39 0.45*		
C	-0.627661000	1.971276000	-1.073263000	*-0.02 -0.51 -0.49*		
O	-1.005985000	3.036623000	-1.248262000	*0.07 -0.30 0.02*		
C	-3.944503000	-0.879210000	0.118978000	0.01 -0.00 -0.00		
H	-4.058334000	-1.879005000	0.560234000	0.00 0.00 0.00		
H	-4.416562000	-0.133221000	0.770236000	0.00 0.00 0.00		
H	-4.434303000	-0.900727000	-0.864357000	0.00 0.00 0.00		
C	0.729916000	-2.544868000	2.963768000	-0.00 -0.00 -0.01		
H	0.901266000	-3.587942000	2.684989000	0.00 0.00 0.00		
H	1.575903000	-2.138632000	3.528051000	0.00 0.00 0.00		
H	-0.160252000	-2.475140000	3.601137000	0.00 0.00 0.00		
C	3.994521000	0.268450000	-0.903262000	-0.01 -0.00 0.00		
H	4.497154000	-0.198645000	-0.044288000	0.00 0.00 0.00		
H	4.321295000	-0.251889000	-1.812502000	0.00 0.00 0.00		
H	4.276317000	1.329073000	-0.932024000	0.00 0.00 0.00		
C	-0.016679000	2.471562000	2.096037000	-0.01 0.00 -0.04		
O	0.013902000	3.164872000	2.973479000	-0.00 -0.01 -0.03		

Table S23. Transition state of CO+O_2 process in presence of 2 more CO molecules absorbed on copper. (doublet)

31				Imaginary frequency (364 cm^{-1})			
Cu	1.325832000	-0.072189000	-0.030478000	-0.07	0.01	-0.03	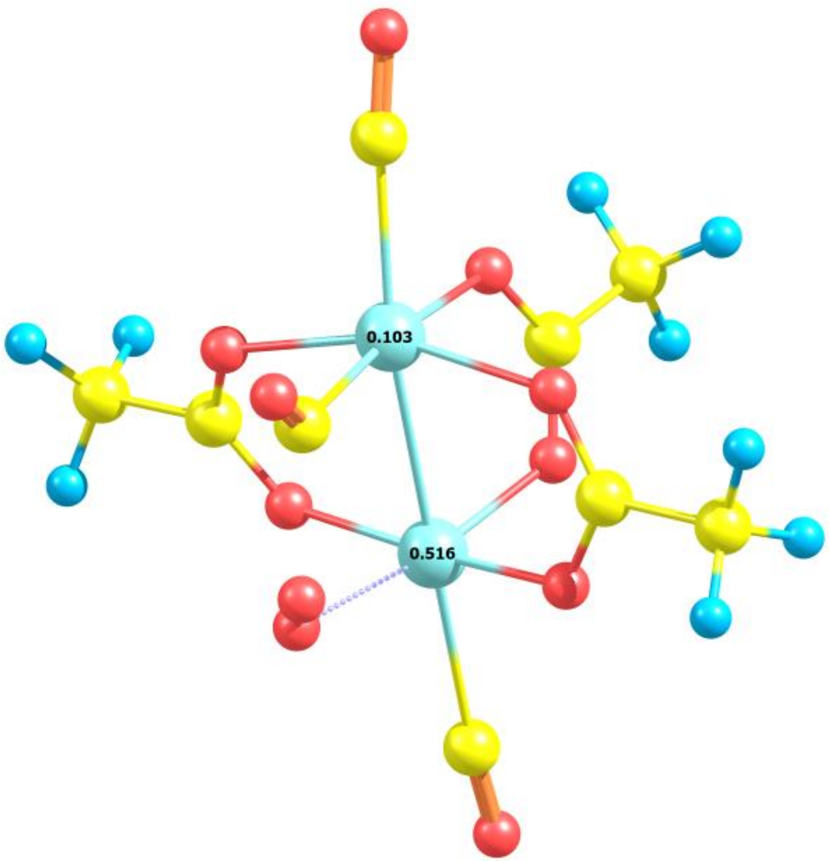
Cu	-1.298082000	0.043643000	-0.166712000	-0.02	0.01	-0.05	
O	-1.235981000	-1.908097000	-0.405901000	0.01	-0.03	0.03	
C	-0.154288000	-2.471514000	-0.703432000	-0.02	0.03	0.01	
O	0.967027000	-1.917759000	-0.797090000	0.01	0.08	0.01	
O	1.074387000	1.909688000	0.398173000	0.02	-0.06	-0.08	
O	0.854966000	-0.646018000	1.865250000	0.02	0.02	-0.02	
C	-0.027843000	2.502609000	0.310026000	-0.01	-0.01	-0.02	
O	-1.121651000	1.981023000	-0.023499000	0.00	0.03	0.02	
O	-1.322573000	-0.145736000	1.787126000	0.01	-0.00	-0.01	
C	-0.272570000	-0.497430000	2.384856000	-0.00	0.01	-0.01	
O	-1.054970000	0.085110000	-2.161826000	0.17	-0.03	0.12	
O	-0.172568000	0.857795000	-2.611363000	0.58	-0.06	0.26	
C	1.559897000	0.616985000	-1.933788000	-0.63	0.05	-0.17	
O	2.415955000	1.040971000	-2.548055000	-0.25	-0.09	0.15	
C	-0.063214000	3.974918000	0.636973000	0.01	-0.01	0.00	
H	-0.681959000	4.119496000	1.532635000	0.00	0.00	0.00	
H	0.944889000	4.364095000	0.815887000	0.00	0.00	0.00	
H	-0.549833000	4.512597000	-0.186625000	0.00	0.00	0.00	
C	-0.383520000	-0.742828000	3.863409000	0.01	0.00	-0.01	
H	-1.431143000	-0.837573000	4.159209000	0.00	0.00	0.00	
H	0.188427000	-1.638815000	4.121896000	0.00	0.00	0.00	
H	0.072230000	0.107686000	4.384668000	0.00	0.00	0.00	
C	-0.219213000	-3.959860000	-0.953401000	0.01	0.01	0.00	
H	-0.216126000	-4.475367000	0.017121000	0.00	0.00	0.00	
H	-1.157240000	-4.203693000	-1.466401000	0.00	0.00	0.00	
H	0.649436000	-4.297163000	-1.528780000	0.00	0.00	0.00	
C	-3.685890000	0.112878000	-0.255727000	0.00	-0.00	0.01	
O	-4.803554000	0.153058000	-0.201866000	0.00	0.00	-0.01	
C	3.434060000	-0.286055000	0.536160000	0.03	0.01	-0.02	
O	4.430469000	-0.450242000	1.018859000	0.01	-0.00	0.00	

Table S24. **IV** - Cyclic Cu^{+}-C(O)-O-O-Cu^{2+} structure, product of **TS-I** (doublet)

27				CO vibration (1963 cm^{-1})		
Cu	-0.037978000	-0.373430000	-1.059073000	0.00	0.00	-0.00
Cu	0.092621000	0.328511000	1.287564000	-0.00	-0.00	0.00
O	2.046847000	0.144054000	1.026720000	0.00	-0.00	-0.00
C	2.452383000	-0.537347000	0.065142000	-0.00	0.00	0.01
O	1.741434000	-0.987115000	-0.889002000	-0.00	-0.00	-0.00
O	-1.920357000	-0.136928000	-0.937324000	0.00	-0.00	-0.00
O	0.393127000	1.555817000	-1.400180000	0.00	0.01	0.01
C	-2.463913000	0.233614000	0.151186000	0.01	0.00	0.01
O	-1.873359000	0.418174000	1.232018000	-0.00	-0.00	-0.01
O	0.203369000	2.185157000	0.739916000	-0.00	0.00	0.00
C	0.387426000	2.414842000	-0.487848000	0.00	-0.01	-0.01
O	0.126068000	-1.579946000	1.616298000	-0.00	-0.01	-0.02
O	-0.670599000	-2.377931000	0.745880000	0.00	0.01	-0.08
C	-0.560318000	-2.222695000	-0.524509000	*0.16*	*0.41*	*0.71*
O	-0.817153000	-2.893519000	-1.447614000	*-0.12*	*-0.32*	*-0.42*
C	-3.943873000	0.442002000	0.091309000	-0.00	0.00	-0.00
H	-4.317639000	0.832449000	1.034133000	-0.00	-0.00	-0.00
H	-4.182122000	1.125465000	-0.723701000	-0.00	0.00	0.00
H	-4.428191000	-0.509185000	-0.133510000	-0.01	0.00	0.00
C	0.646197000	3.840733000	-0.870169000	-0.00	0.00	0.00
H	0.168403000	4.522319000	-0.169227000	0.00	0.01	-0.00
H	1.723436000	4.015060000	-0.827824000	-0.00	0.00	0.00
H	0.312789000	4.031425000	-1.888282000	-0.00	-0.00	0.00
C	3.904635000	-0.892526000	0.006602000	0.00	0.00	-0.00
H	4.260922000	-0.884526000	-1.021750000	0.00	0.00	0.00
H	4.488240000	-0.216719000	0.627159000	0.01	-0.00	-0.00
H	4.019274000	-1.907459000	0.392773000	0.00	0.00	0.00

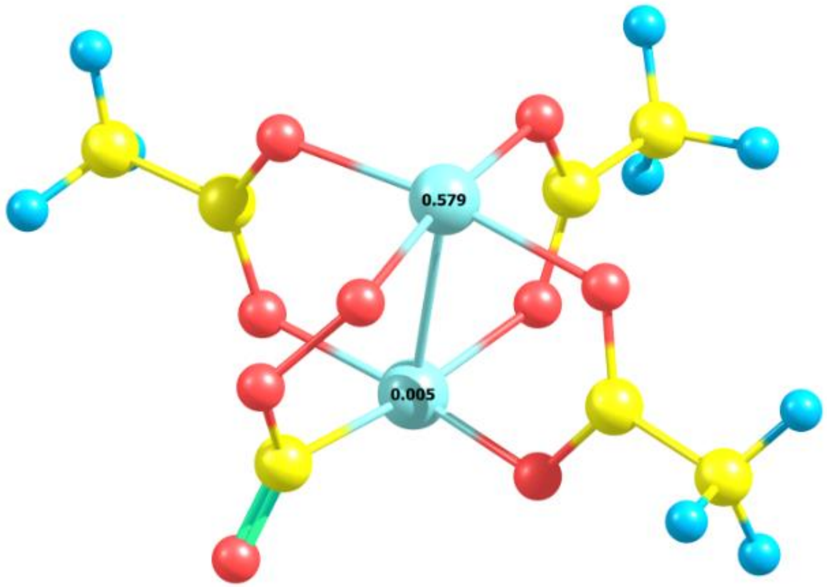

Table S25. **V** – CO-attachment to **IV**, on the “oxygen” side. (doublet)

29			
Cu	-0.418369000	1.126934000	0.711485000
Cu	0.333190000	-0.841974000	-0.614871000
O	2.126205000	0.061343000	-0.364317000
C	2.184365000	1.266372000	-0.063915000
O	1.207682000	1.993101000	0.307256000
O	-2.176459000	0.412998000	0.702414000
O	0.237265000	0.053408000	2.265486000
C	-2.420192000	-0.679188000	0.100169000
O	-1.600507000	-1.340561000	-0.562093000
O	0.624043000	-1.797918000	1.068555000
C	0.572183000	-1.148039000	2.148216000
O	0.086532000	0.417366000	-2.081978000
O	-0.968740000	1.366495000	-1.984757000
C	-1.108133000	2.048669000	-0.903294000
O	-1.663154000	3.049240000	-0.655547000
C	-3.830669000	-1.168945000	0.206832000
H	-4.483206000	-0.485093000	-0.338228000
H	-3.924714000	-2.169471000	-0.207212000
H	-4.147259000	-1.155255000	1.249396000
C	0.974299000	-1.878615000	3.394724000
H	0.855919000	-2.953262000	3.273045000
H	2.028874000	-1.670370000	3.587315000
H	0.402671000	-1.518633000	4.248257000
C	3.503549000	1.969944000	-0.149696000
H	3.630334000	2.646354000	0.693978000
H	4.318445000	1.251682000	-0.196549000
H	3.509817000	2.572109000	-1.060360000
C	1.074413000	-2.504209000	-1.998325000
O	1.415744000	-3.306421000	-2.699105000

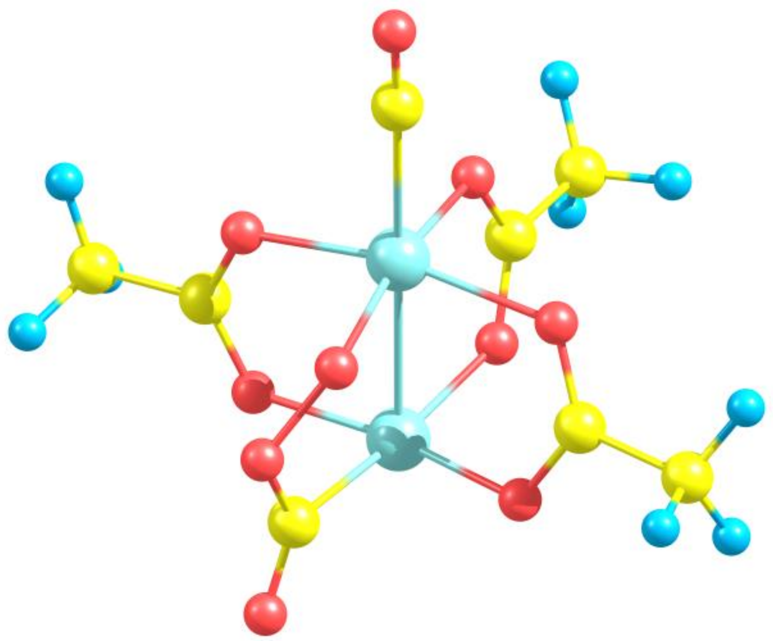

Table S26. **TS-II** (doublet)

29				Imaginary frequency (570 cm^{-1})		
Cu	0.128913000	0.367649000	1.312948000	-0.00	-0.01	-0.01
Cu	-0.036138000	-0.105500000	-1.115458000	-0.01	-0.03	-0.01
O	-1.890745000	0.542055000	-0.951196000	0.00	0.00	0.01
C	-2.374409000	0.755730000	0.181957000	0.00	-0.00	-0.01
O	-1.762211000	0.644365000	1.283410000	0.01	0.01	0.01
O	1.844237000	-0.473405000	1.248452000	-0.01	0.01	0.01
O	0.686272000	2.181302000	0.572259000	-0.01	-0.03	0.01
C	2.394347000	-0.729669000	0.138428000	-0.00	0.00	-0.01
O	1.847377000	-0.664193000	-0.984158000	-0.00	0.00	0.01
O	0.533946000	1.668671000	-1.600496000	0.00	0.00	0.00
C	0.771734000	2.458569000	-0.640802000	-0.00	-0.01	-0.01
O	-0.619478000	-1.930596000	-0.440224000	*0.00*	*-0.02*	*0.74*
O	-0.707204000	-2.300054000	1.153300000	0.02	0.06	-0.19
C	-0.437734000	-1.473773000	2.026247000	0.07	0.24	-0.12
O	-0.377264000	-1.329359000	3.187422000	-0.04	-0.12	-0.06
C	-0.707311000	-2.117964000	-2.375439000	*0.06*	*0.22*	*-0.50*
O	-0.985647000	-2.962644000	-3.060914000	-0.04	-0.12	-0.02
C	3.840084000	-1.114854000	0.199772000	-0.00	0.00	0.00
H	4.043103000	-1.631768000	1.133677000	0.00	0.00	0.00
H	4.125109000	-1.727143000	-0.654283000	0.00	0.00	0.00
H	4.439201000	-0.200365000	0.193662000	0.00	0.00	0.00
C	1.160824000	3.854658000	-1.019503000	-0.00	-0.00	0.01
H	1.728596000	3.867533000	-1.948013000	0.00	0.00	0.00
H	0.246553000	4.430475000	-1.170399000	0.00	0.00	0.00
H	1.721459000	4.315509000	-0.211613000	0.00	0.00	0.00
C	-3.816725000	1.147221000	0.276634000	0.00	-0.00	0.00
H	-3.966599000	1.827774000	1.112746000	0.00	0.00	0.00
H	-4.180238000	1.587958000	-0.650623000	0.00	0.00	0.00
H	-4.386771000	0.239079000	0.481026000	0.00	0.00	0.00

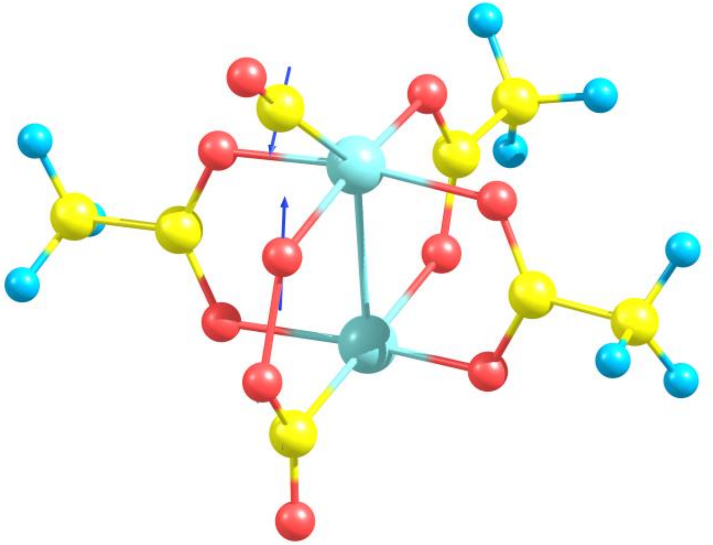

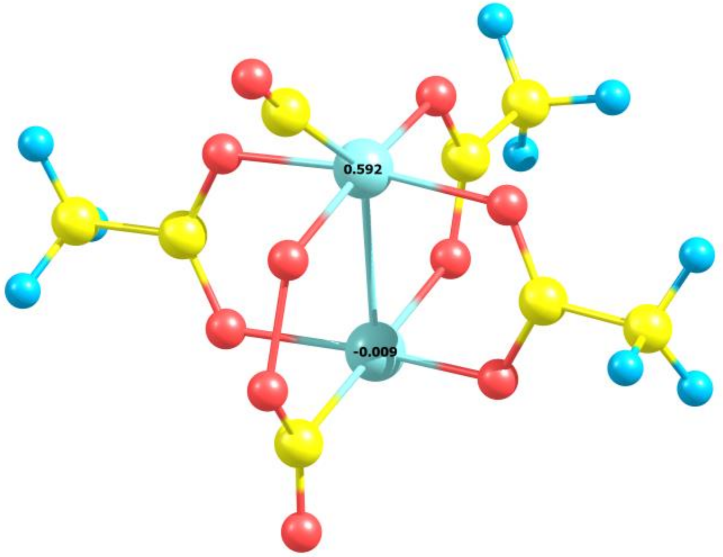

Table S27. Transition state of CO – O2 exchange on Pristine PW.

34				Imaginary frequency ($19\ cm^{-1}$)		
O	1.440932500	-0.595077000	-2.047754000	-0.01	0.00	-0.00
C	1.644732000	0.637476000	-1.915516000	-0.00	0.00	0.00
O	1.186465000	1.370980000	-1.002800000	0.00	0.00	0.00
Cu	0.431698000	-1.560277000	-0.681166000	-0.00	0.00	0.00
Cu	-0.085442000	0.660559500	0.310701000	-0.00	0.01	0.00
O	-1.504078500	0.756716000	-1.043142500	0.01	-0.00	-0.01
C	-1.796888000	-0.245883500	-1.744492000	0.00	-0.01	-0.00
O	-1.197528500	-1.349291500	-1.742829000	-0.01	-0.01	0.00
O	2.014047000	-1.583602500	0.464681000	-0.00	0.01	-0.00
O	-0.622385000	-2.332751000	0.771190000	0.00	-0.00	0.00
C	2.153321000	-0.702519000	1.349025000	-0.00	0.00	0.00
O	1.401009000	0.287728000	1.534441500	-0.00	0.00	0.01
O	-1.297221000	-0.324760000	1.501372000	-0.00	-0.00	-0.00
C	-1.295700000	-1.582153000	1.520754000	0.00	-0.00	-0.00
C	3.312705000	-0.844816000	2.289361000	-0.00	0.00	-0.00
H	4.009182000	-1.604154000	1.942522000	0.00	0.00	0.00
H	3.815318000	0.114780000	2.406541000	0.00	0.00	0.00
H	2.928935000	-1.133480000	3.269306000	0.00	0.00	0.00
C	-2.199594000	-2.251534000	2.512374000	-0.00	-0.00	0.00
H	-2.421487000	-1.586861000	3.344342000	0.00	0.00	0.00
H	-3.135362000	-2.502288000	2.008368000	0.00	0.00	0.00
H	-1.755331000	-3.180742000	2.864665000	0.00	0.00	0.00
C	-2.950759000	-0.095537000	-2.689611000	-0.00	-0.00	-0.00
H	-3.693976000	0.590594000	-2.286369000	0.00	0.00	0.00
H	-2.572110000	0.326094000	-3.622845000	0.00	0.00	0.00
H	-3.393781000	-1.063677000	-2.910977000	0.00	0.00	0.00
C	2.508536000	1.311398000	-2.939409000	-0.00	0.00	0.00
H	2.941038000	0.590449000	-3.628705000	0.00	0.00	0.00
H	1.905598000	2.034339000	-3.490977000	0.00	0.00	0.00
H	3.296259000	1.869401000	-2.433964000	0.00	0.00	0.00
O	-3.832520996	2.108475504	0.896511501	*0.38*	*0.41*	*0.07*
C	-2.739565995	2.327320002	1.039271500	*0.44*	*0.16*	*-0.01*
O	0.433561505	3.162667002	1.352719501	*0.43*	*0.18*	*0.05*
O	1.555533005	3.562998000	1.410518999	*0.48*	*0.04*	*-0.08*

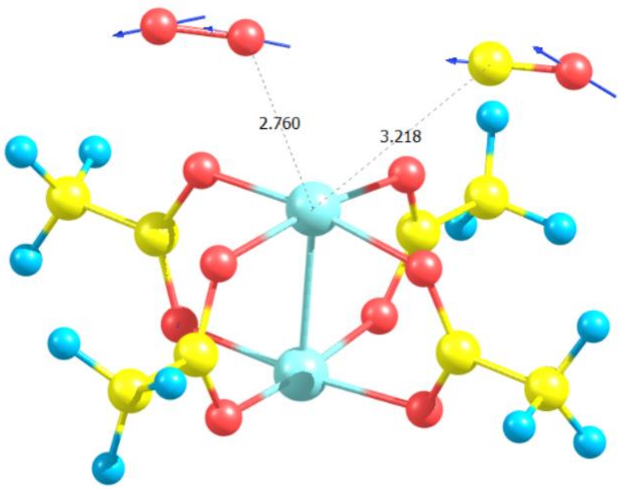

Table S28. CASSCF electronic structures, examples of optimization and singlepoint calculations

CAS(3*3)/def2-SVP – **TS-I**

```
---------------------------------------------
CAS-SCF STATES FOR BLOCK  1 MULT= 4 NROOTS= 1
---------------------------------------------

ROOT   0:  E=   -4220.8901706412 Eh
      1.00000 [     0]: 111


---------------------------------------------
CAS-SCF STATES FOR BLOCK  2 MULT= 2 NROOTS= 1
---------------------------------------------

ROOT   0:  E=   -4220.8901582765 Eh
      0.41037 [     0]: 210
      0.39637 [     6]: 012
      0.19326 [     3]: 111


-----------------------------
SA-CASSCF TRANSITION ENERGIES
------------------------------

LOWEST ROOT (ROOT 0 ,MULT 4) =  -4220.890170641
Eh -114856.261 eV

STATE   ROOT MULT  DE/a.u.    DE/eV    DE/cm**-1
   1:    0    2   0.000012     0.000      2.7
```

CAS(5*4)/def2-TZVP – **TS-I**

```
---------------------------------------------
CAS-SCF STATES FOR BLOCK  1 MULT= 4 NROOTS= 1
---------------------------------------------

ROOT   0:  E=   -4222.4643503453 Eh
      1.00000 [     3]: 2111


---------------------------------------------
CAS-SCF STATES FOR BLOCK  2 MULT= 2 NROOTS= 1
---------------------------------------------

ROOT   0:  E=   -4222.4643437760 Eh
      0.40860 [    15]: 2210
      0.39529 [     9]: 2012
      0.19611 [    12]: 2111


-----------------------------
SA-CASSCF TRANSITION ENERGIES
------------------------------

LOWEST ROOT (ROOT 0 ,MULT 4) =  -4222.464350345
Eh -114899.096 eV

STATE   ROOT MULT  DE/a.u.    DE/eV    DE/cm**-1
   1:    0    2   0.000007     0.000      1.4
```

```
-----------------------------
 NEVPT2 TRANSITION ENERGIES
------------------------------

LOWEST ROOT (ROOT 0, MULT 2) =  -4226.792933213
Eh -115016.883 eV

STATE ROOT MULT  DE/a.u.     DE/eV    DE/cm**-1
   1:   0    4   0.000048     0.001     10.6
```

CAS(5*4)/def2-TZVP – **IV**

```
---------------------------------------------
CAS-SCF STATES FOR BLOCK  1 MULT= 4 NROOTS= 1
---------------------------------------------

ROOT   0:  E=   -4222.4249274859 Eh
      0.99890 [     3]: 2111


---------------------------------------------
CAS-SCF STATES FOR BLOCK  2 MULT= 2 NROOTS= 1
---------------------------------------------

ROOT   0:  E=   -4222.4653475823 Eh
      0.77629 [    15]: 2210
      0.22167 [     9]: 2012


-----------------------------
SA-CASSCF TRANSITION ENERGIES
------------------------------

LOWEST ROOT (ROOT 0 ,MULT 2) =  -4222.465347582
Eh -114899.123 eV

STATE   ROOT MULT  DE/a.u.    DE/eV    DE/cm**-1
   1:    0    4   0.040420     1.100   8871.2
```

```
                   89        90        91
               -0.41622  -0.23987  -0.02382
                1.63071   1.00011   0.36935
               --------  --------  --------
 0 Cu dx2y2       41.9       0.0      33.4
 0 Cu dxy         10.1       0.0       8.1
 1 Cu dx2y2        0.0      86.6       0.1
13 C  py          10.3       0.0      15.1
14 O  py           6.5       0.0       5.7
```

CASSCF(3*3) **TS-I** optimisation

```
! def2-SVP OptTS Freq
! TightSCF
%geom
 Calc_Hess true # Calc. Hessian in beginning
 Recalc_Hess 5  # Recalc. Hessian 5 steps
end
%casscf
 nel 3 # number of active electrons
 norb 3 # number of active orbitals
 mult 4,2 # multiplicity blocks
end
```

NEVPT2(5*4)/def2-TZVP singlepoint

```
! def2-TZVP
! TightSCF
%casscf
 nel 5 # number of active electrons
 norb 4 # number of active orbitals
 mult 4,2 # multiplicity blocks
 NEVPT2 SC
End
```

Table S29. Orbitals 88-91 of CASSCF. These 4 orbitals with 5 electrons have to be included in active space of structures **III** and **TS-I**

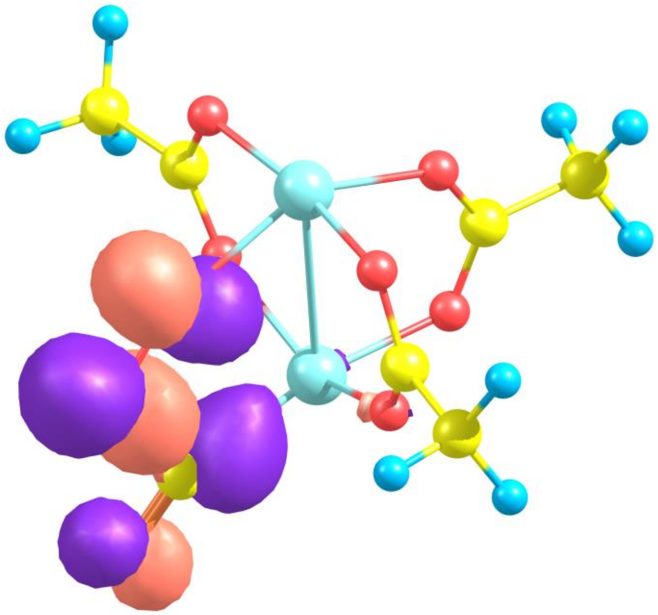	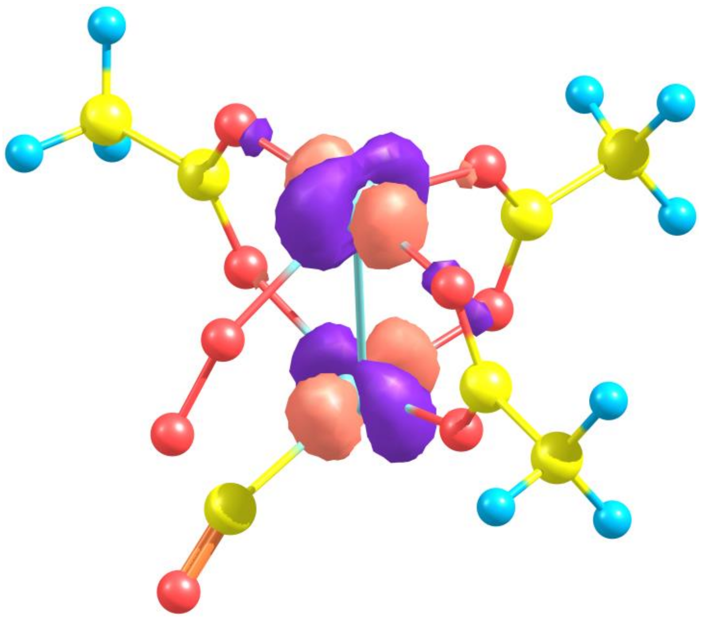
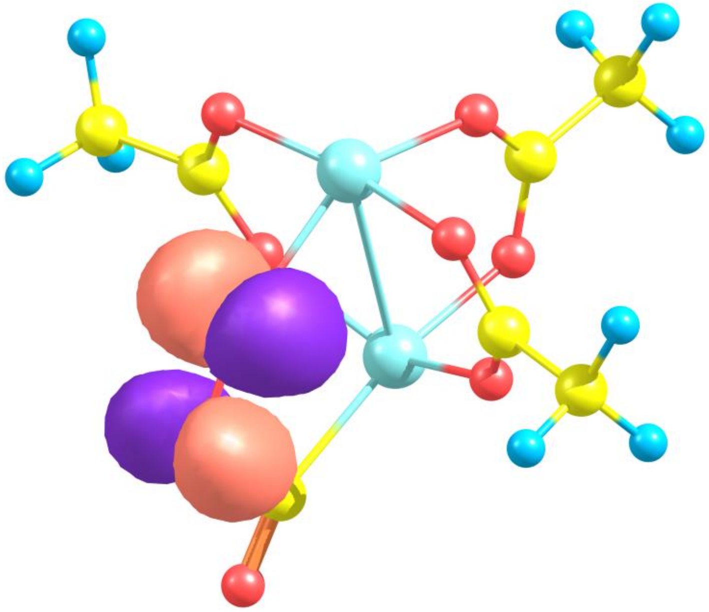	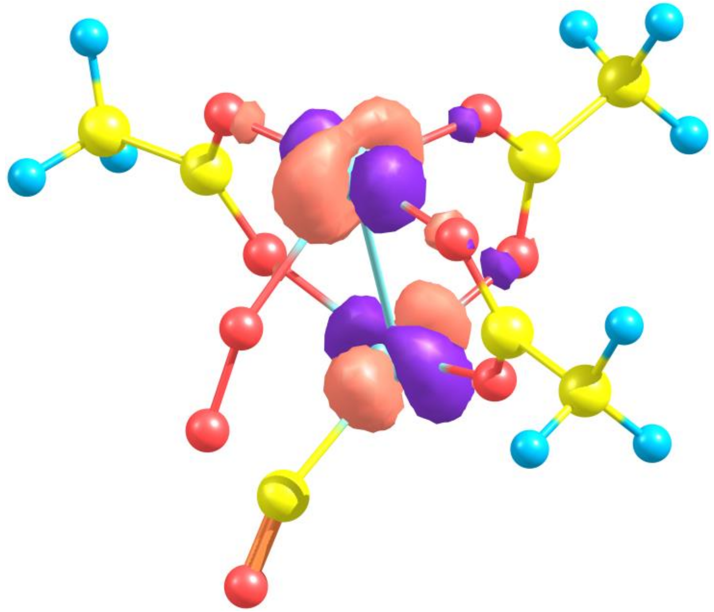
